# Efficient Polarization‐Entangled Photon‐Pair Generation by a Fiber‐In‐Line van der Waals Material

**DOI:** 10.1002/advs.76712

**Published:** 2026-07-17

**Authors:** Jungseok Choi, Seongju Ha, Seungjae Lim, Joohyeon Ahn, Jaekyoung Kim, Jong Hyuk Yim, Nam Hun Park, Youngdong Yoo, Jae‐Ung Lee, Hee Su Park, Sang Min Lee, Dong‐Il Yeom

**Affiliations:** ^1^ Department of Energy Systems Research Ajou University Suwon Gyeonggi‐do Republic of Korea; ^2^ Korea Research Institute of Standards and Science (KRISS) Daejeon Republic of Korea; ^3^ Department of Chemistry Ajou University Suwon Gyeonggi‐do Republic of Korea; ^4^ Department of Physics Ajou University Suwon Gyeonggi‐do Republic of Korea; ^5^ Agency for Defense Development (ADD) Daejeon Republic of Korea

**Keywords:** all‐fiber devices, entangled photons, quantum materials, spontaneous parametric down‐conversion

## Abstract

The recent emergence of van der Waals (vdW) materials with exceptional optical nonlinearity has driven advances in next‐generation nonlinear photonic devices beyond classical optics. While integrating these materials into fiber‐optic platforms offers unprecedented functionalities in quantum systems, realizing telecom‐band in‐line photon‐pair sources based on highly nonlinear vdW materials remains challenging. Here, we report photon‐pair generation centered at telecom wavelength via spontaneous parametric down‐conversion in a fiber‐integrated device incorporating a ferroelectric vdW crystal, SnP_2_S_6_. Benefiting from its giant optical nonlinearity and relaxed phase‐matching across near‐visible to telecom‐band wavelengths, a remarkable nonlinear conversion efficiency of 0.07% is achieved in a few‐micrometer‐thick SnP_2_S_6_ film. Moreover, the fiber‐integrated SnP_2_S_6_ device delivers high‐quality photon pairs at telecom wavelengths, achieving a detected coincidence rate of 102 counts/s. The coincidence‐to‐accidental ratio reached up to 55 662, which is much larger than that of previous vdW‐material‐based sources. Furthermore, the device generates polarization‐entangled photon pairs, which are verified via quantum state tomography to exhibit fidelities of 0.97 and concurrences of 0.95. This demonstration establishes a scalable, robust fiber‐integrated platform that combines the versatility of vdW materials, paving the way toward low‐loss, functionalized quantum photonic systems for networking, sensing, and computing.

## Graphical abstract

Telecom‐wavelength polarization‐entangled photon pairs are generated in a van der Waals‐material‐integrated all‐fiber device via spontaneous parametric down‐conversion. The demonstrated fiberized quantum light source bridges emerging 2D nonlinear materials and practical quantum photonic systems, opening new opportunities for scalable fiber‐based quantum technologies.

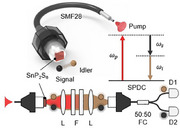

## 1 Introduction

Efficient sources of quantum‐mechanically correlated photon pairs are a key element in quantum technologies. From fundamental proof of quantum mechanics [1, 2, 3] to realization of advanced quantum information processing apparatuses [4, 5], quantum optical processes based on spontaneous parametric down‐conversion (SPDC) or spontaneous four‐wave mixing (SFWM) in nonlinear optical media have long served as the cornerstone for generating photonic quantum states. The general advantages of such nonlinear‐optical sources include access to well‐defined spectro‐temporal modes and the possibility of flexible control over the operating wavelengths. In particular, photon pairs covering a large wavelength bandwidth benefit high‐capacity and multiple‐node wavelength‐division multiplexed quantum communications [6, 7], high‐resolution quantum metrology [8], and entanglement‐based efficient imaging at long wavelengths reaching the THz range [9, 10]. It is worth noting that the monumental Hong‐Ou‐Mandel experiment using SPDC also mentions accurate time‐interval measurement relying on a large bandwidth as a main application [11]. However, advancements in this direction require materials research to maximize nonlinear effects, since extensive propagation through bulk nonlinear media generally restricts the photon bandwidth due to phase‐matching constraints. Thin and small‐scale‐integrable photon pair sources thereby become a critical component in photonic quantum information processing, offering universal applicability across diverse wavelength requirements.

Extensive research has revealed that van der Waals (vdW) materials, while advantageous for scalable integration systems due to their ultrathin‐ness and robustness, possess superior optical nonlinearity compared to that of conventional crystals [12, 13, 14]. Crucially, unlike bulk crystals, vdW materials are subject to significantly relaxed phase‐matching conditions [14]. Leveraging these unique properties, recent studies have increasingly focused on broadband photon‐pair generation in various vdW materials via SPDC [15, 16, 17, 18, 19, 20]. The first breakthrough vdW material to demonstrate SPDC was NbOCl_2_ [15]. This material exhibits ferroelectricity alongside exceptionally weak interlayer interactions, enabling a monolayer‐like excitonic resonance, resulting in outstanding second‐order optical nonlinearity even at multilayer configurations [15]. Subsequently, quantum‐correlated photon pairs were successfully demonstrated in 3R‐MS_2_ (M═Mo, W) and rhombohedral boron nitride (rBN) [16, 19, 20]. These achievements mark a notable step toward realizing miniaturized quantum photonic devices and circuits using vdW materials.

Direct fiber integration of quantum light sources is highly desirable for compatibility with quantum network systems. Conventional all‐fiber photon‐pair sources have been mostly implemented using the relatively weak SFWM process, since the primary nonlinear response of amorphous silica optical fiber is third‐order Kerr nonlinearity [21, 22, 23, 24]. To address this, there have been tremendous efforts to manifest second‐order optical nonlinearity by integrating vdW materials in various fiber platforms, including tapered fiber, side‐exposed‐core fibers, and photonic crystal fibers, resulting in significant advancement in enhancing the nonlinear second harmonic signal generated up to two orders of magnitude compared to that of the monolayer vdW material [25, 26, 27]. Recently, a fiber‐integrated SPDC source based on the twisted vdW crystal stack was reported [28], where the photon pairs via SPDC process was generated at the wavelength of around 810 nm with moderate purity and coincidence rates.

Here, we demonstrate telecom‐wavelength polarization‐entangled photon‐pair generation via SPDC using a vdW‐material‐integrated fiber device. A SnP_2_S_6_ ferroelectric material, belonging to the *R_3_* space group with broken inversion symmetry, is employed as a vdW material exhibiting strong nonresonant second‐order nonlinearity [29, 30, 31, 32, 33, 34]. Thickness‐dependent second‐harmonic generation (SHG) analysis reveals that the nonlinear coherence length, *L_c_*, of SnP_2_S_6_ material reaches several micrometers thanks to the small index difference *Δn* between pump and SHG wavelengths. A remarkably high SHG conversion efficiency of 0.07% was obtained in 2.7 µm‐thick SnP_2_S_6_ for a pump laser at a telecom wavelength, which is comparable to or higher than that of recently reported periodically poled layered semiconductor [35]. A SnP_2_S_6_ thin film was transferred onto a standard optical fiber (SMF28e, Corning) facet to perform SPDC experiments where high‐quality photon pairs with 102 counts per second (cps) were successfully detected for a fiber‐delivered 782 nm continuous‐wave (CW) pump laser with an optical power of 36 mW. The maximum coincidence‐to‐accidental ratio (CAR) was measured to be 55 662 at a pump power of 0.7 mW, which is much larger than the maximum CAR values reported for previous vdW‐material‐based SPDC sources [15, 16, 17, 18, 19, 20, 28]. Moreover, to reveal the polarization entanglement generation capability of our source, we generated two Bell states, |Φ^−^〉 and |Ψ^+^〉, by pump‐polarization tuning and post‐selection. The reconstructed density matrices (ρ’s) by quantum state tomography (QST) display that the generated quantum states are in excellent agreement with the ideal states, exhibiting fidelities up to 0.97, purities up to 0.97, and concurrences exceeding 0.95. Our result of SPDC in a fiber‐integrated vdW material would be a significant step for the development of in‐line quantum light sources compatible with a plug‐and‐play quantum network system. Moreover, we believe that the realization of all‐fiber high‐dimensional quantum states or a compact all‐fiber quantum interferometer would be feasible with various optical fiber platforms combining vdW materials based on our results.

## 2 Results

### 2.1 Material Characterization of SnP_2_S_6_ Thin Film

**FIGURE 1:**
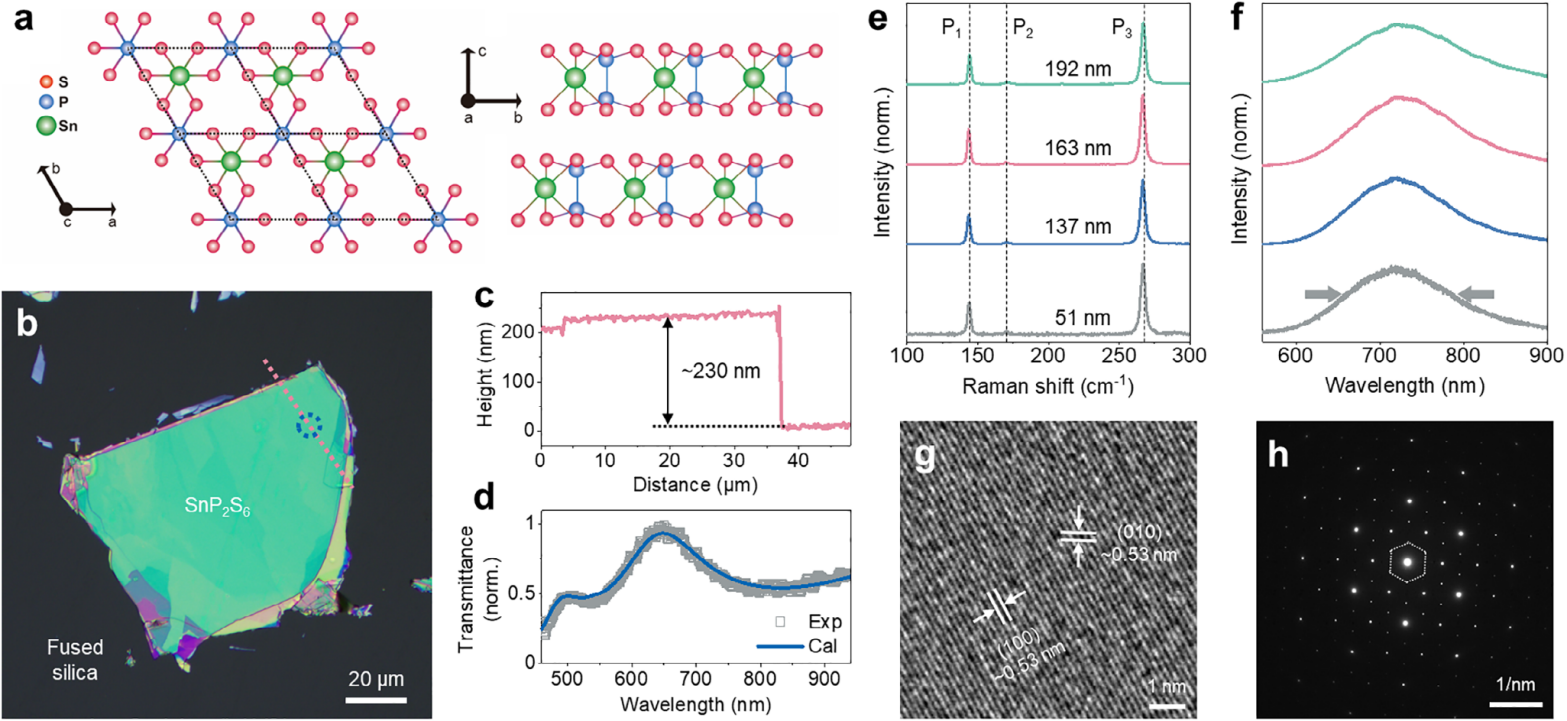
Thickness analysis and optical responses in SnP_2_S_6_ thin films. (a) Top (left) and side (right) views of the atomic structure of SnP_2_S_6_, which consists of vdW force‐stacked layers and belongs to the *R_3_* space group. (b) Optical microscope image of a mechanically exfoliated SnP_2_S_6_ flake on a 1 mm‐thick fused silica substrate. (c) Height profile of SnP_2_S_6_ flake along the pink‐dotted line in (b), measured by AFM technique. (d) Normalized linear optical transmittance of the region of SnP_2_S_6_ flake marked by the blue‐dotted circle in (b). The gray square and blue solid line represent experimental data and the calculated curve for a thickness of 232 nm, respectively. (e,f) Normalized Raman and photoluminescence spectra from SnP_2_S_6_ samples with various thicknesses pumped by a 532 nm CW laser. Dark‐field transmission electron microscopy image (g) and corresponding selected‐area electron diffraction pattern (h) of an exfoliated SnP_2_S_6_ thin film.

SnP_2_S_6_ has a rhombohedral crystal structure (Figure 1a), belonging to the *C_3_* point group symmetry and the *R_3_* space group, which incorporates translational symmetry [32, 33, 34]. Atoms are strongly combined by covalent and ionic bonds within a layer, and layers are stacked by weak vdW forces [33] as depicted in Figure 1a. Samples were prepared via a mechanical exfoliation method typically used for vdW materials. Figure 1b shows an optical microscope image of an SnP_2_S_6_ flake exfoliated from a bulk crystal (HQ graphene) onto a 1 mm‐thick fused silica substrate. The thickness of the samples was measured both by using atomic force microscopy (AFM) and by fitting the transmittance spectrum to thickness, refractive index, and extinction coefficient as proposed by Swanepoel [36]. Figure 1c is the height profile along the pink dotted line in Figure 1b, measured by AFM, revealing a thickness of 230 nm for the SnP_2_S_6_ flake. The normalized optical transmittance (gray squares), measured from the blue dotted circle in Figure 1b, aligns well with the Swanepoel calculation (blue curve), with thickness being the sole fitting parameter. These results confirmed the reliability of the Swanepoel method for determining the thickness of the samples (Figure S1). We next investigated the Raman and photoluminescence (PL) characteristics of SnP_2_S_6_ films using a 532 nm CW laser (Figure 1e,f, and Figure S2). Normalized data were used for straightforward comparison. Three distinct peaks, P_1_ (∼143 cm^−1^), P_2_ (∼170 cm^−1^), and P_3_ (∼267 cm^−1^), were observed in the Raman spectra, which are known to be intrinsic phonon modes of SnP_2_S_6_, arising from internal stretching vibrations of the S‐P‐S bond [30, 32, 33]. The positions of these peaks showed no observable change under our measurement conditions, even as the thickness increased from 51 nm to several micrometers (Figures S1 and S2). Despite its indirect bandgap, SnP_2_S_6_ is known to emit PL across a broad spectral range due to the formation of self‐trapped excitons induced by atomic structural distortion [32]. All measured samples exhibited a wide PL with a full‐width at half‐maximum (FWHM) of approximately 150 nm, spanning 600–900 nm (indicated by gray arrows in Figure 1f). While the spectral shape of PL was modified by interference effects at greater thicknesses, the fundamental emission range remained largely unaffected (Figure S2). The observed insensitivity of both Raman peak positions and emission characteristics of PL against thickness variations is consistent with previous works, which is attributed to the weak interlayer interaction within SnP_2_S_6_ [30, 32, 33, 37]. In addition, we verified the single‐crystalline structure and composition ratio of the exfoliated SnP_2_S_6_ thin film via transmission electron microscopy and energy dispersive X‐ray spectroscopy techniques (Figure 1g,h, and Figure S3) [32].

### 2.2 Wavelength & Thickness Dependence of SHG in SnP_2_S_6_

**FIGURE 2:**
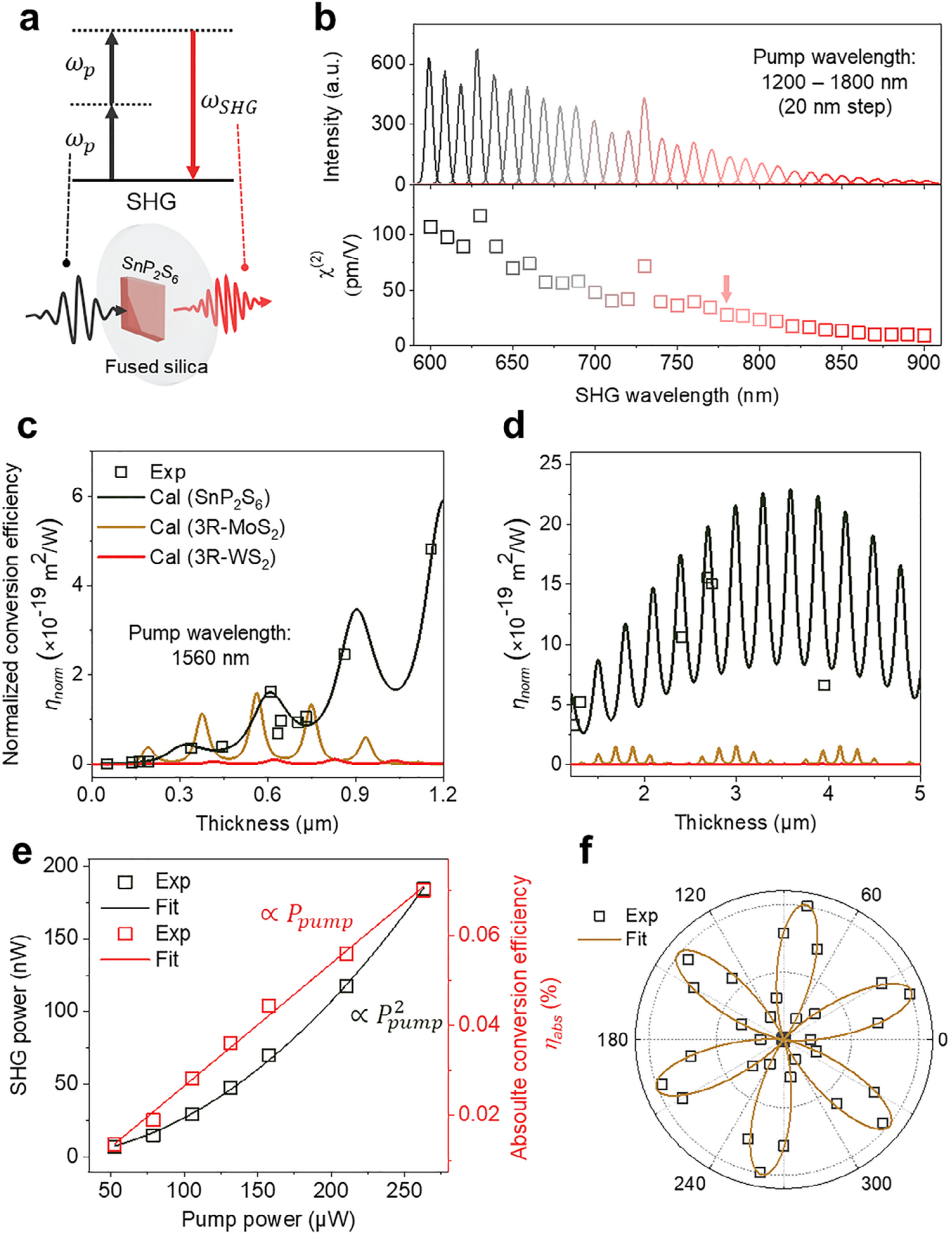
Nonlinear optical characterization of SnP_2_S_6_ thin films. (a) Schematic diagrams of the SHG process (upper) and a SnP_2_S_6_ thin film on a fused silica substrate (lower). Here, ω_*p*(*SHG*)_ represents the angular frequency of the pump (second‐harmonic) light. (b) Broadband SHG spectra (upper) and estimated effective second‐order nonlinear susceptibility χ^(2)^ (lower) of a 14 nm‐thick SnP_2_S_6_ sample. Thickness‐dependent normalized conversion efficiency of SHG (defined as $\eta_{norm}=I_{SHG}\;/I_{p}^{2}$) for SnP_2_S_6_ thin films with thicknesses less than (c) and greater than (d) 1.2 µm, respectively, for clarity. Black squares represent the experimental data, while colored solid lines show the results of theoretical calculations for SnP_2_S_6_ (black), 3R‐MoS_2_ (yellow), and 3R‐WS_2_ (red). For the calculations, complex refractive indices and phase‐matching conditions were considered together with χ^(2)^ values of 28, 91, and 16.2 pm/V for SnP_2_S_6_, 3R‐MoS_2_, and 3R‐WS_2_, respectively [32, 37, 38, 39]. (e) Pump power‐dependence of absolute SHG power (black) and conversion efficiency (defined as η_*abs*_ = *P_SHG_* /*P_pump_*; red) for a 2.7 µm‐thick SnP_2_S_6_ sample. (f) Polarization‐resolved SHG pattern exhibiting six‐fold rotational symmetry. In (e,f), colored squares and solid lines denote experimental data and fitting curves, respectively. The center wavelength of the SHG for (c–f) was 780 nm, corresponding to the downward red arrow in (b).

We conduct broadband SHG spectroscopy to probe the second‐order nonlinearity of SnP_2_S_6_ using an optical parametric amplifier (OPA) system (Figure S4 and Experimental Section) [40]. This technique directly measures the frequency doubling of incident pump photons, as shown in Figure 2a. The upper graph of Figure 2b displays the SHG spectra for a 14 nm‐thick SnP_2_S_6_ sample for the pump wavelength range of 1200–1800 nm with 20 nm intervals. The SHG intensity gradually increased as the pump wavelength decreased. We attribute this trend to a two‐photon resonance effect, which occurs when the SHG photon energy approaches the bandgap of a material [12, 13, 14, 41]. Using the SHG spectra, we estimate the broadband χ^(2)^ of SnP_2_S_6_ based on the nonlinear Maxwell's equations (Experimental Section) [41, 42]. The calculated χ^(2)^ values, shown in the lower graph of Figure 2b, ranged from 9 to 117 pm/V. These values are comparable to those previously reported [33, 34], demonstrating the strong potential of SnP_2_S_6_ for broadband nonlinear optical applications.

To better understand the properties of SnP_2_S_6_ for practical implementation, we investigated the thickness dependence of SHG intensity in our samples using a 1560 nm pump beam within the optical communication C‐band (Figure 2c,d). We analyzed the SHG intensity in terms of normalized conversion efficiency η_*norm*_, defined as $I_{SHG}/I_{p}^{2}$ (in units of m^2^/W), which allows us to mainly focus on the effects of the material's thickness. Our experimental data (black squares) showed excellent agreement with the theoretical η_*norm*_ (black solid line), which was calculated based on the nonlinear Maxwell's equations (Experimental Section) with the estimated χ^(2)^ value of 28 pm/V (indicated by the red arrow in Figure 2b). The efficiency η_*norm*_ exhibits a long‐period envelope and superimposed short‐period oscillations as a function of thickness. The long‐period envelope is governed by the nonlinear coherence length *L_c_*, while the short‐period oscillations are caused by the Fabry–Pérot interference at the two film boundaries [35, 38]. The calculated *L_c_*, which is defined as π/Δ*k* with Δ*k* being the wavenumber mismatch was 3.5 µm for SnP_2_S_6_ [41]. It should be noted that this value is several times greater than those of 3R‐MoS_2_ (*L_c_* ∼590 nm; yellow solid line) and 3R‐WS_2_ (*L_c_* ∼813 nm; red solid line), both of which are actively studied for their strong second‐order nonlinearity (Figure 2c,d) [37, 38, 39, 40]. Given that SHG intensity scales with the square of the interaction length, SnP_2_S_6_ potentially achieves an order of magnitude greater η_*norm*_ than 3R‐MS_2_.

We then proceeded to measure the SHG power as a function of pump power for the 2.7 µm‐thick SnP_2_S_6_ thin film, which displayed the highest η_*norm*_ among all samples (Figure 2e). As expected, the SHG power exhibited a clear quadratic dependence on the pump power (black squares and solid lines) [41]. We also calculated the absolute conversion efficiency η_*abs*_, defined as *P_SHG_*/*P_p_*, which represented a predictable linear dependence on pump power (red squares and solid line) [41]. Impressively, the sample recorded a maximum η_*abs*_ of 0.07% at a pump power of 260 µW (peak power of 69 GW/cm^2^), which is well above the η_*abs*_ of 0.03% reported for a 3.4 µm‐thick 3R‐MoS_2_ with 3 poling periods at a higher pump power of 52 mW (peak power of 127 GW/cm^2^) [35]. A polarization‐resolved SHG response shows a 6‐fold SHG pattern (Figure 2f), clearly reflecting the structural symmetry of SnP_2_S_6_ (Figure 1h), consistent with the previous works [33, 34].

### 2.3 SHG of Optical Fiber Device Integrated With SnP_2_S_6_

**FIGURE 3:**
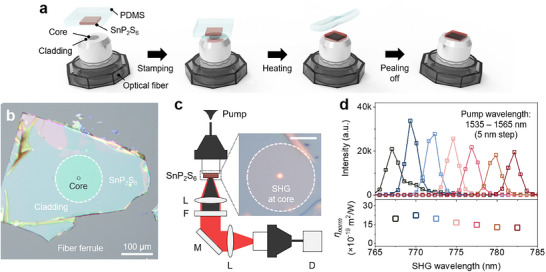
Fabrication and SHG responses of SnP_2_S_6_‐integrated optical fiber. (a) Schematic illustration of the PDMS‐assisted transfer process of SnP_2_S_6_ onto the optical fiber. (b) Optical microscope image of a SnP_2_S_6_ thin film uniformly transferred onto the end‐facet of a fiber ferrule. The large white (small black) dashed circle indicates the border of the fiber cladding (core). (c) Schematic diagram of the SHG measurement setup for the SnP_2_S_6_‐integrated optical fiber. L: aspheric lens, F: short‐pass filter, M: mirror, D: detector. Inset: Optical microscope image of an SHG spot at the fiber core, pumped by our OPA source centered at 1350 nm. (d) SHG spectra from optical fiber‐integrated SnP_2_S_6_, pumped by a tunable C‐band CW laser with 5 nm steps from 1535 to 1565 nm (upper). The average pump power was fixed at 30 mW. The lower panel shows the normalized SHG conversion efficiency, corresponding to the spectra in the upper panel.

We fabricate an SnP_2_S_6_‐integrated optical fiber device first through a PDMS‐assisted transfer process (Figure 3a). The process begins by using an optical microscope to align the SnP_2_S_6_ thin film, which is adhered to a transparent PDMS substrate, with the core of a commercially available SMF28 fiber equipped with an FC/PC ferrule. The film is then carefully attached to the fiber surface with a high‐precision XYZ stage. The PDMS is separated from the film through a subsequent heating and peeling‐off process. Figure 3b shows that the SnP_2_S_6_ thin film was uniformly transferred across a large area of the integrated optical fiber, including both the core and cladding regions. Upon launching a 1350 nm‐centered pulse from the OPA source, we clearly observed an SHG spot localized at the fiber core (inset of Figure 3c). The experimental setup shown in Figure 3c was configured to measure the SHG response from the SnP_2_S_6_‐integrated optical fiber (Experimental Section). The upper graph of Figure 3d displays the SHG spectra from our device, obtained by adjusting the CW pump wavelength from 1535 to 1565 nm in 5 nm steps. SHG responses with a CW laser followed the same trend as the broadband results obtained with ultrafast pulses from an OPA source (Figure 2b). The bottom graph of Figure 3d represents the conversion efficiency η_*norm*_, derived from the data at the pump wavelengths. The values of η_*norm*_ were consistent with those measured from the SnP_2_S_6_ thin film on a transparent substrate (Figure 2c,d). To further elaborate on the long‐term stability and reproducibility of the fabricated devices, the SHG responses were monitored over 7 days, demonstrating stable operation without notable degradation of performance (Figure S5). These observations verify that the SnP_2_S_6_ thin film was transferred onto the fiber ferrule without quality degradation, enabling strong interaction with light guided along the fiber core.

### 2.4 SPDC in SnP_2_S_6_‐Integrated Optical Fiber at Telecom Bands

**FIGURE 4:**
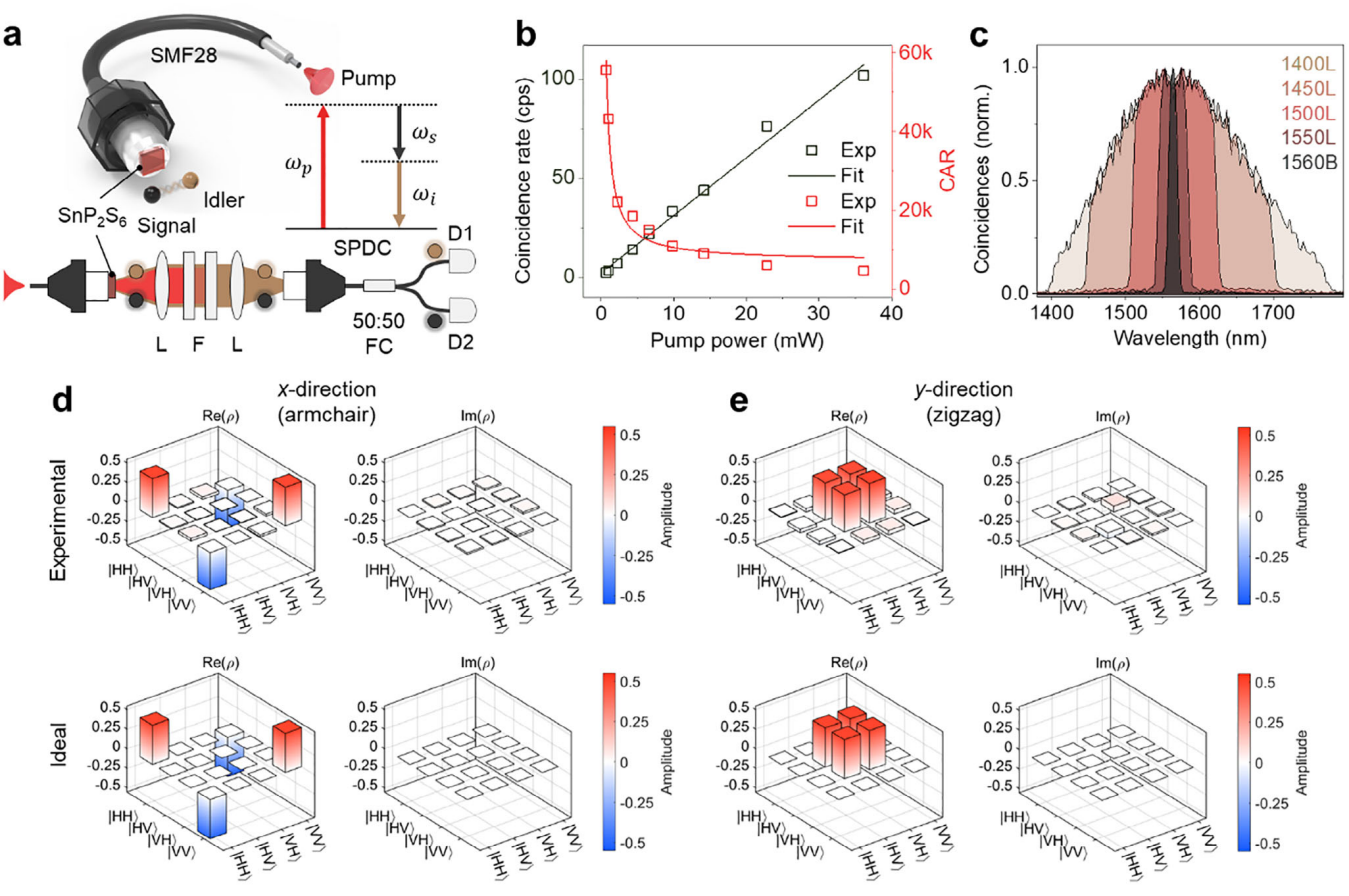
Measurement results for polarization‐entangled photon pairs from SnP_2_S_6_‐integrated optical fiber. (a) Structures of the SnP_2_S_6_‐integrated optical fiber (top), SPDC process (middle), where ω_*p*(*s*, *i*)_ represents the angular frequency of the pump (signal, idler), and experimental setup with a fiber‐type Hanbury Brown‐Twiss interferometer (bottom). L: aspheric lens, F: spectral filter, FC: fiber coupler, D: detector. (b) Power‐dependent average coincidence rate (black) and CAR (red) measured for 100 s of integration time. (c) Spectra of SPDC from the SnP_2_S_6_ film measured by fiber spectroscopy. Spectral filters are noted as 1XX0L for LPFs, while 1560B indicates a bandpass filter centered at 1560 nm with a FWHM of 12 nm. (d,e) The experimentally reconstructed (top) and the ideal Bell‐state (bottom) density matrices, generated under a pump polarization aligned with the crystallographic *x*‐direction (d) and *y*‐direction (e) of the SnP_2_S_6_ film.

Efficient generation of quantum‐correlated photon pairs can be achieved fundamentally via SPDC in highly second‐order nonlinear media [43]. To explore the SPDC capabilities of a 2.7 µm‐thick, fiber‐integrated SnP_2_S_6_ film noted for its high SHG efficiency (Figures 2d and 3d), we pumped it using a 782 nm CW laser as depicted in the upper diagrams in Figure 4a. The generated photon pairs were measured using a fiber‐based Hanbury Brown‐Twiss (HBT) interferometer, which quantifies photon correlation by recording the temporal coincidence counts (the bottom diagram in Figure 4a and the Experimental Section). Figure S6a shows the pump‐power‐dependent coincidence counts from our SnP_2_S_6_‐integrated optical fiber, obtained using a long‐pass filter (LPF) with a 1500 nm cut‐on wavelength, isolating photon pairs within the telecommunication S‐ to U‐band due to the energy‐conservative parametric process. A sharp peak appeared near the zero‐delay time, a distinct feature unobservable in the bare fiber under various pump power and filtration conditions (Figure S6a). A close examination of the narrow temporal range showed that these counts were well‐fitted by a Gaussian curve, where the FWHM, representing the overall timing resolution, remained reasonably constant between 182 and 188 ps regardless of the pump power (Figure S6b). To clarify the origin of these coincidences, we analyzed the power‐law scaling of both the single‐channel detection rate and coincidence counts, as established by comparing the 1400 and 1500 nm LPFs (see Note S1 and Figure S7b,c). While the single‐channel counts exhibit a sub‐linear scaling, mainly stemming from material fluorescence undergoing power‐dependent saturation, the coincidence counts scale linearly with a unity slope, which is a characteristic feature of a typical SPDC process [15, 16, 17, 18, 19, 20]. Thus, this observation suggests that the acquired coincidence counts primarily originate from the SPDC process, without being significantly influenced by the single‐channel background noise.

As key performance indicators for SPDC sources, the coincidence rate and coincidence‐to‐accidental ratio (CAR) are central to assessing the generation efficiency and photon‐pair purity, respectively [43]. The coincidence rate was calculated by dividing the net coincidence counts (after accidental subtraction) by the integration time, while the CAR was defined as the ratio of this rate to the average accidental count rate (Experimental Section) [43]. Figure 4b presents these metrics as a function of pump power, measured with a 1500 nm LPF. The data clearly demonstrate that the coincidence rate scales linearly with pump power, whereas the CAR is inversely proportional, reflecting the typical properties of the SPDC process [15, 16, 17, 18, 19, 20, 43, 44]. The maximum coincidence rate reached 101.6 cps at 36 mW of pump power, corresponding to a brightness of 2.8 cps per mW. Moreover, benefiting from the well‐defined spatial mode of the optical fiber, a CAR value exceeding 55 000 was achieved at a pump power of 0.7 mW, as shown in Figure 4b. This value represents a substantial enhancement of 1–4 orders of magnitude compared to the maximum CAR values reported for previously demonstrated vdW‐material‐based sources under their respective measurement conditions (Table S1) [15, 16, 17, 19, 20, 28, 35], underscoring the superior correlated photon purity of our fiber‐integrated quantum light source. Leveraging the experimentally obtained CAR values, we quantitatively evaluated the nonclassicality of the generated photon pairs by the Cauchy–Schwarz inequality with the violation parameter $R={\left(g_{si}^{\left(2\right)}\right)}^{2}\;/g_{ss}^{\left(2\right)}g_{ii}^{\left(2\right)}\leq 1$, where classical fields must satisfy this boundary [45, 46]. Here, $g_{si}^{\left(2\right)}$ denotes the normalized cross‐correlation function between signal and idler photons, while $g_{ss}^{\left(2\right)}$ and $g_{ii}^{\left(2\right)}$ represent the autocorrelation functions of the individual signal and idler channels, respectively. Under the CW pumping condition, the cross‐correlation value obtained at zero‐delay time corresponds to CAR + 1 (Ref. 15). Given the multimode characteristics of our broadband SPDC source ($1\leq g_{ss}^{\left(2\right)},g_{ii}^{\left(2\right)}\leq 2$) [47, 48], *R* is determined to be within the range of 7.75 × 10^8^ ≤ *R* ≤ 3.10 × 10^9^. This value exceeds the classical limit by over eight orders of magnitude, thus verifying the nonclassical nature of our fiber‐integrated source. Additionally, the temporal stability under continuous pumping was examined by tracking the coincidence counts over a 4 h measurement period (Figure S8). As shown in the figure, the coincidence histograms maintained a low background level over time, reflecting high photon purity and highlighting the feasibility of our fiber device for stable generation of entangled photons.

To investigate the spectral profile of the generated photon pairs, we conducted dispersive‐fiber spectroscopy using a 1 km‐long SMF‐28 fiber spool and compared it with the calculation of the corresponding SPDC spectra for the fiber‐tip/2.6 µm‐thick SnP_2_S_6_/air multilayer structure (Figure 4c and Figure S9 and Notes S2 and S3) [49]. When the wavelength difference between the signal and idler photons becomes large, the group‐velocity mismatch increases, leading to a larger delay time after passing through the dispersive medium, which in turn broadens the spectral width for the coincidence measurements [49]. The measured and calculated SPDC spectra showed reasonable agreement regarding the expected photon‐pair bandwidth depending on various filtration conditions, encompassing various optical telecommunication bands, which is attributed to the relaxed phase‐matching condition arising from the small *Δn* between interacting photons [33]. The maximum spectral width over 370 nm was measured using the LPF with a 1400 nm cut‐on wavelength. It is noteworthy that the value and shape of SPDC spectra were primarily limited by the bandwidth of the 50:50 fiber coupler (±100 nm) and the detector's quantum efficiency (optimized at 1550 nm) in our experiment [49].

We further examined the coincidence rate and CAR for various spectral filters at the fixed pump power of 16.1 mW. The upper graph in Figure S10 shows that the coincidence rate increased as the cut‐on wavelength of the LPF was shortened, thereby broadening the photon‐pair bandwidth. The experimental results are well fitted by the calculated SPDC spectra in our SnP_2_S_6_ film. Conversely, the CAR demonstrated an inverse dependence on the cut‐on wavelength of the LPF (lower graph in Figure S10). This trend is primarily attributed to increased background noise responses (Figure S7 and Note S1) originating from the material with shorter cut‐on wavelengths [15, 20, 35]. When a 1560 nm bandpass filter with a FWHM of 12 nm was employed under the same pump conditions, the measured coincidence rate closely followed the JSI prediction, further confirming the spectral consistency of the SPDC process. In this narrowband regime, the CAR reached 41,196, illustrating that narrower‐bandwidth filtering effectively suppresses broadband noise.

For an independent verification of the nonclassicality of our source, we additionally performed the QST analysis to characterize its polarization entanglement. Given that SnP_2_S_6_ belongs to the *R*_3_ space group, the non‐vanishing second‐order susceptibility tensor components satisfy $\chi_{xxx}^{\left(2\right)}=-\;\chi_{xyy}^{\left(2\right)}=-\;\chi_{yxy}^{\left(2\right)}=-\chi_{yyx}^{\left(2\right)}$ and $-\;\chi_{yxx}^{\left(2\right)}=\chi_{yyy}^{\left(2\right)}=-\;\chi_{xxy}^{\left(2\right)}=-\chi_{xyx}^{\left(2\right)}$ [33]. Since $\chi_{xxx}^{\left(2\right)}$ is more than an order of magnitude greater than $\chi_{yyy}^{\left(2\right)}$, the 6‐fold polarization‐resolved SHG pattern in Figure 2f confirms the dominant tensor component, and directly indicates the crystallographic *x*‐ and *y*‐directions along the armchair and zigzag axes, respectively (Figure 1a) [33, 34]. Accordingly, the polarization‐entangled photon‐pair state as a function of the pump polarization angle $\phi_{p}$ relative to the *x*‐direction is given by $\left|\psi \right\rangle \approx \frac{\cos(\phi_{p})}{\sqrt{2}}\left(\left|HH\rangle -|VV\right\rangle \right)-\frac{\sin(\phi_{p})}{\sqrt{2}}\left(\left|HV\rangle +|VH\right\rangle \right).$ An *x*‐polarized pump generates the $|\Phi^{-}\rangle =1/\sqrt{2}\left(\left|HH\rangle -|VV\right\rangle \right)$ state, whereas a *y*‐polarized pump yields the $|\Psi^{+}\rangle =1/\sqrt{2}\left(\left|HV\rangle +|VH\right\rangle \right)$ state. For experimental validation, the photon pairs were split via a nonpolarizing beam‐splitter for QST using 16 measurement bases, defined by quarter‐wave plates (QWPs), half‐wave plates (HWPs), and linear polarizers (LPs) (Figure S11a). Coincidence counts were accumulated for 50 s per each basis using combinations of the horizontal (*H*), vertical (*V*), anti‐diagonal (*A*), right‐circular (*R*), and left‐circular (*L*) polarization states for both input polarization states with identical pump power of 11 mW (Figure S11b,c). The two‐photon polarization‐state density matrices ρ’s were reconstructed by maximum likelihood estimation (MLE; Figure 4d,e) [50]. The reconstructed quantum states showed excellent agreement with the ideal Bell states. The *x*‐polarized pump yielded a fidelity of 0.97 ± 0.02 (≈ |Φ^−^〉), a purity of 0.94 ± 0.03, and a concurrence of 0.95 ± 0.02. For the *y*‐polarized pump, the fidelity, purity, and concurrence were 0.97 ± 0.03 (≈ |Ψ^+^〉), 0.97 ± 0.05, and 0.97 ± 0.03, respectively. These results demonstrate that our SnP_2_S_6_ fiber‐integrated device functions as a high‐quality polarization‐entangled photon‐pair source, capable of selectively generating distinct Bell states depending on the pump polarization direction. These entanglement metrics are also summarized in Table S1, along with a comparison with prior vdW quantum light sources.

Finally, as a proof‐of‐concept of an all‐fiber configuration without any free‐space coupling/imaging optics, we built a compact in‐line SPDC experimental setup as shown in Figure S12a,b. A series of conventional isolators at the telecom wavelength was used as an in‐line pump laser blocker, which provided an extinction ratio of 70 dB at 782 nm while exhibiting high transmittance across a wide range of telecommunication wavelengths (Figure S12c). Although the overall performance is slightly degraded (possibly due to sample deformation during the fiber connection and isolator losses), a relatively high coincidence rate (61.2 cps) and CAR (12,011) were maintained (Figure S12d–f). This finding potentially suggests the feasibility of an alignment‐free SPDC generation and detection system in fully integrated all‐fiber configurations, paving the way toward compact and practical quantum light sources compatible with existing fiber networks and sensing systems.

## 3 Conclusion

In summary, we have demonstrated the generation of high‐CAR quantum‐correlated photon pairs at telecom wavelength using a SnP_2_S_6_‐integrated standard optical fiber device. Together with the large second‐order optical nonlinearity over the near‐infrared spectral range and relaxed phase‐matching conditions, we could obtain an absolute SHG conversion efficiency up to 0.07% in a 2.7 µm‐thick SnP_2_S_6_ multilayer film. The SnP_2_S_6_ multilayer film transferred onto the fiber ferrule also shows a high η_*norm*_ of 15 × 10^−19^ m^2^/W, when pumped by a tunable C‐band CW laser, indicating the efficient nonlinear light‐matter interaction within the optical fiber platform. We have conducted the SPDC experiment using a pump centered at 782 nm, with which the photon pair generation through the SPDC process was observed at telecom wavelengths in a fiber‐optic integrated device. A maximum CAR reached 55,662 at a CW pump power of 0.7 mW with a 1500 nm long‐pass filter, which is much larger than that reported in previous works. Furthermore, we confirmed that the polarization‐entangled photon pairs, arising from the intrinsic crystal symmetry of the material, exhibit high state fidelity, purity, and concurrence. A preliminary all‐fiber structure showed promising photon‐pair generation capability proving remarkable compatibility with all‐fiber configurations. It should be highlighted that these results were obtained merely in a bare SnP_2_S_6_ thin film, indicating the huge potential for further enhancement of nonlinear and quantum optical figure‐of‐merits via material engineering techniques, such as periodic poling or resonant structure employment [17, 35, 51]. Our alignment‐free all‐fiber scheme further opens the possibility of realizing an in‐line compact quantum nonlinear interferometer [52]. Convincingly, the combination of the novel quantum/nonlinear material developments with advanced specialty optical fiber technologies paves the way toward practical and reliable quantum technologies, enabling low‐loss transmission of high‐dimensional quantum information [53].

## 4 Experimental Section

### 4.1 Broadband SHG Spectroscopy and Linear Optical Transmittance

We examined the second‐order nonlinearity of SnP_2_S_6_ thin film using a transmission‐type broadband SHG spectroscopy setup, based on an OPA system with a 200 fs pulse duration τ and 200 kHz of repetition rate *f_rep_* (Figure S4) [40]. The pump power was controlled by a half‐wave plate (HWP) and a polarization beam splitter. The pump beam was focused onto the sample by the objective lens (OL) and then blocked by a short‐pass filter (SPF) after inducing SHG. The SHG light from the sample was collimated by a second OL, collected by a 550 µm‐core multi‐mode fiber via a fiber coupler, and measured with a highly sensitive TE‐cooled spectrometer (Ocean Optics, QE pro). For polarization‐resolved measurement (Figure 2f), the polarization angle of the incident pump beam was adjusted using an HWP placed before the focusing OL together with the rotational linear polarizer in front of the spectrometer.

Additionally, for linear optical transmittance measurements (Figure 1d and Figure S1), we used a flippable beam splitter to locate a supercontinuum (SC) source along the same beam path as the pump and SHG beams. The transmitted SC beam was collimated by a second OL and measured with the same spectrometer used for the SHG spectroscopy.

### 4.2 Estimation of Broadband Effective Second‐Order Nonlinearity χ^(2)^ and SHG Power

For the estimation of χ^(2)^ of SnP_2_S_6_, we begin with the calibration of our spectrometer using SHG responses from monolayer MoS_2_ and WS_2_, which are van der Waals materials with well‐known nonlinear characteristics [12, 13, 14, 38, 40]. Because photon energy range in our experiments was below the bandgap of SnP_2_S_6_, we used the expression of SHG power *P_SHG_* based on the nonlinear Maxwell's equation for a non‐absorptive material as [41, 42]:

$$\;P_{SHG}=\frac{2{\left|\chi^{\left(2\right)}\right|}^{2}\omega_{p}^{2}P_{p}^{2}}{n_{p}^{2}n_{SHG}\varepsilon_{0}c^{3}}\;\frac{L^{2}}{f_{rep}\tau W^{2}{\left[\frac{\pi }{4\ln2}\right]}^{3/2\;}}\sin c^{2}\left(\frac{\Delta kL}{2}\right)$$

where *c* is the speed of light, ε_0_ is the vacuum permittivity, *L* is the thickness of SnP_2_S_6_ thin film, *n_p_* and *n_SHG_* are the refractive indices at the pump and SHG wavelengths [33], respectively, Δ*k* is the phase‐mismatch component (wavenumber mismatch), *W* is the spot size of the focused pump beam, and *P_p_* is the pump power.

### 4.3 Characterization of SHG and SPDC Responses in SnP_2_S_6_‐Integrated Optical Fiber

To measure the SHG response from our SnP_2_S_6_‐integrated optical fiber device, we employed a fiber‐coupled, tunable C‐band CW laser as the pump source. The pump light exiting the fiber went through a fiber polarization controller (FPC) and was then incident on the sample. The pump and generated second‐harmonic waves were then collimated by a lens and passed through an SPF, which blocked the strong residual pump light. The filtered SHG was then refocused into a multi‐mode fiber via another lens and measured by a spectrometer. The polarization state of the pump beam entering the sample was controlled by the FPC and was verified to be parallel to the optical table by using a free‐space linear polarizer placed just after the fiber device.

For the SPDC measurement, the experimental setup was partially reconfigured. The tunable C‐band laser was replaced with a fiber‐coupled CW laser centered at 782 nm. To block the strong residual pump light, a long‐pass filter with an edge wavelength of 1100 nm was used in front of the spectral filters. The multi‐mode fiber was replaced with an SMF28 optical fiber‐based HBT interferometer, equipped with the superconducting nanowire single‐photon detector (Quantum Opus), which converted incident photons into electrical pulses with a detection efficiency over 80% at 1550 nm. These pulses were then recorded by a time tagging module (Swabian Instruments, Time Tagger Ultra). For the quantitative evaluation of the photon‐pair characteristics, the coincidence analysis was performed by acquiring the full correlation histogram over a 40 ns range with 1‐ps time‐bins. The detailed data‐processing procedures for extracting net coincidence counts, along with control experiments for background noise contributions and power‐law scaling analysis, are described in Figure S6a,c and S7, and Note S1.

The total photon‐pair detection efficiency (η_*total*_) of our measurement system is estimated as $\eta_{total}=T_{opt}^{2}\;\times \eta_{coupl}^{2}\times \eta_{BS}\times \eta_{PD}^{2}\times \eta_{band}^{2}$. For the free‐space configuration, the individual parameters are assigned as follows: bulk optics transmission *T_opt_* = 0.99 × 0.99 × 0.93 × 0.92 (accounting for the aspheric lens, 1100/1500 nm LPFs, and 50:50 fiber beam‐splitter and fiber polarization controller (FPC) insertion losses), single‐mode fiber coupling efficiency η_*coupl*_ = 0.75, probabilistic beam‐splitting factor η_*BS*_ = 0.5, and SNSPD quantum efficiency η_*PD*_ = 0.8 at 1550 nm. Incorporating a bandwidth factor of η_*band*_ = 0.5 based on [49], η_*total*_ is estimated to be 3.2%. Similarly, for the all‐fiber configuration, *T_opt_* becomes 0.34 considering four in‐line telecom band isolators, a 50:50 fiber beam splitter, and an FPC. η_*band*_ was unity owing to the absence of bandwidth filtering, and the values for η_*BS*_ and η_*PD*_ are retained from the free‐space configuration. Here, to prevent structural damage to the integrated material, the fiber ferrule junction was intentionally not fully tightened, resulting in a fiber‐to‐fiber coupling efficiency (η_*coupl*_) below its typical value of 0.93. Consequent η_*total*_ for the all‐fiber system was evaluated to be 3.2%.

For the dispersive‐fiber spectroscopy, we inserted a 1 km‐long SMF28 fiber spool in front of the 50:50 fiber coupler [49]. The full mathematical calibration procedures for the time‐delay‐to‐wavelength mapping, the wavelength‐dependent dispersion *D*(λ) calculations, and the effective spectral resolution determination based on timing jitter uncertainty are systematically presented in Note S2.

### 4.4 Calculation of SPDC Spectra

The joint spectral intensity (JSI) of photon pairs and the propagation through the SPDC source structure were numerically modeled and calculated to theoretically validate the broadband nature of the fabricated device. To incorporate Fabry–Pérot resonance effects, the transmittance *T*(λ) for the end‐face of the fiber/SnP_2_S_6_/air multilayer was explicitly calculated using the standard Transfer Matrix Method. The complete set of governing equations, including the wavevector mismatch (Δ*k_z_*), Sellmeier equation parameters, and numerical integration models for the filtered JSI, are explained in Note S3.

### 4.5 QST Characterization

The polarization‐entangled state was characterized via two‐qubit QST across 16 measurement bases (Figure S11). The signal and idler photons were separated by a nonpolarizing beam‐splitter and individually projected onto each of a tomographically complete polarization basis set using a sequence of QWP, HWP, and LP. The photons were then coupled into optical fibers via aspheric lenses and detected by SNSPDs. Coincidence counts within a time window of 500 ps were integrated for 50 s per basis. The physical density matrix ρ was reconstructed via MLE [50], from which the state fidelity, purity (Tr(ρ^2^)), and concurrence were evaluated [49, 54]. Uncertainties were determined using an error propagation formula assuming Poisson counting statistics ($\sigma_{N_{i}}=\sqrt{N_{i}}$) [50].

## Author Contributions

**Hee Su Park**, **Sang Min Lee**, and **Dong‐Il Yeom** conceived and supervised the research. **Jungseok Choi** and **Seongju Ha** contributed equally to this work. Jungseok Choi, **Seungjae Lim**, **Jaekyoung Kim**, **Jong Hyuk Yim**, and **Jae‐Ung Lee** characterized the sample and fabricated the optical fiber device. Jungseok Choi, Seongju Ha, and **Nam Hun Park** built the setup and performed the SHG and SPDC experiments. **Joohyeon Ahn** and **Youngdong Yoo** performed electron‐beam‐based microscopy. Jungseok Choi carried out the material thickness analysis. Jungseok Choi and Seongju Ha analyzed the data and wrote the original draft. All authors discussed the results and edited the paper.

## Conflicts of Interest

The authors declare no conflicts of interest.

## Supporting information




**Supporting File**: advs76712‐sup‐0001‐SuppMat.docx.

## Data Availability

The data that support the findings of this study are available from the corresponding author upon reasonable request.
