# Supplementary material for: Efficient Polarization‐Entangled Photon‐Pair Generation by a Fiber‐In‐Line van der Waals Material
Source: Adv Sci (Weinh). 2026 Jul 17:e76712. Online ahead of print. doi: 10.1002/advs.76712 (PMC13379212; doi:10.1002/advs.76712)
Supplement: Supplementary file 1 — Supporting File: advs76712‐sup‐0001‐SuppMat.docx. [file ADVS-9999-e76712-s001.docx]

Supporting Information for

**Efficient Polarization-Entangled Photon-Pair Generation by a Fiber-in-Line van der Waals Material**

Jungseok Choi^1,a^, Seongju Ha^2,a^, Seungjae Lim^1^, Joohyeon Ahn^1,3^, Jaekyoung Kim^1^, Jong Hyuk Yim^1,4^, Nam Hun Park^2,5^, Youngdong Yoo^3^, Jae-Ung Lee^1,4^, Hee Su Park^2,^*, Sang Min Lee^2,^*, and Dong-Il Yeom^1,4,^*

^1^Department of Energy Systems Research, Ajou University, Suwon, Gyeonggi-do 16499, Republic of Korea

^2^Korea Research Institute of Standards and Science (KRISS), Daejeon 34113, Republic of Korea

^3^Department of Chemistry, Ajou University, Suwon, Gyeonggi-do 16499, Republic of Korea

^4^Department of Physics, Ajou University, Suwon, Gyeonggi-do 16499, Republic of Korea

^5^Agency for Defense Development (ADD), Daejeon 34186, Republic of Korea

^a^These authors contributed equally to this work.

^*^Corresponding authors

Hee Su Park: [hspark@kriss.re.kr](mailto:hspark@kriss.re.kr), Sang Min Lee: [samini@kriss.re.kr](mailto:samini@kriss.re.kr), Dong-Il Yeom: [diyeom@ajou.ac.kr](mailto:diyeom@ajou.ac.kr)

**Supporting Figures**


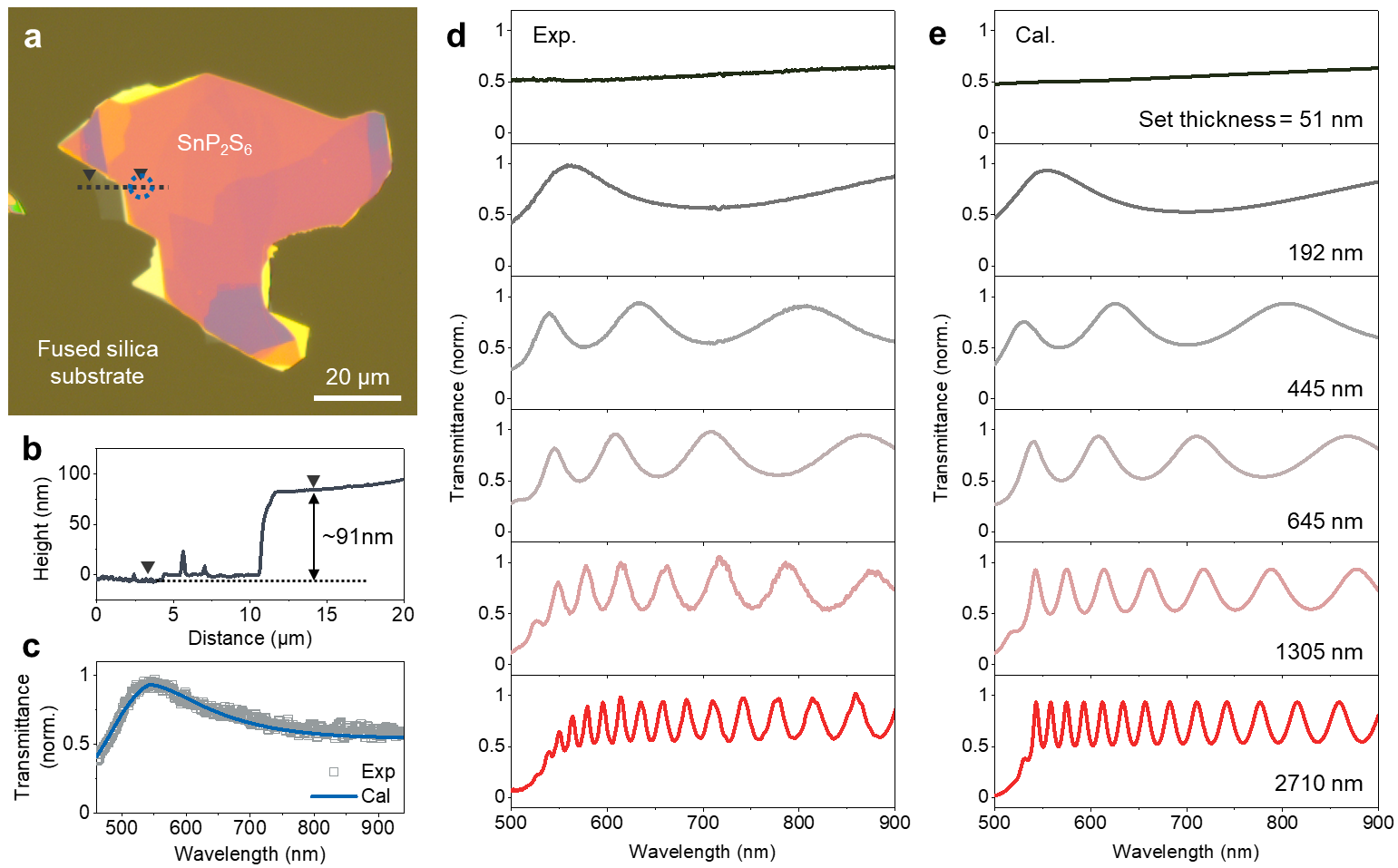


**FIGURE S1** Thickness analysis of SnP_2_S_6_ samples. (a) Optical microscope image of SnP_2_S_6_ flake on a 1 mm-thick fused silica substrate. (b) Height profile of SnP_2_S_6_ flake along the black-dotted line in (a), measured by atomic force microscopy technique. (c) Normalized linear optical transmittance of the region of SnP_2_S_6_ flake marked by a blue-dotted circle in (a). The gray square and blue solid line represent experimental data and the calculated curve via the Swanepoel method with a set thickness of 91 nm, respectively. Experimental (d) and calculated (e) normalized transmittance data for various samples. In (e), the Swanepoel method was applied using thicknesses from 51 nm to 2710 nm.


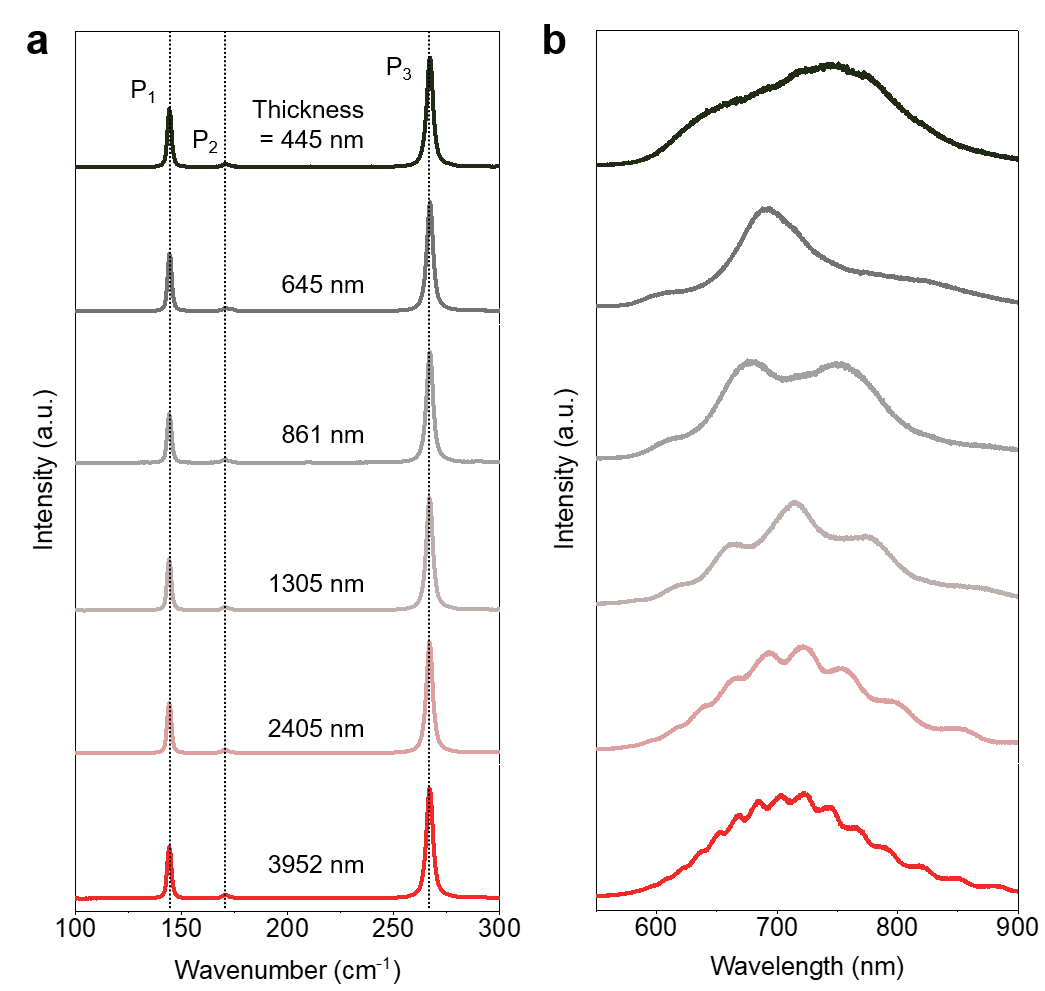


**FIGURE S2** Raman and photoluminescence (PL) spectra in SnP_2_S_6_ samples with various thicknesses. (a) Raman spectra showing three distinct peaks at ~143, ~170, and ~267 cm^-1^, corresponding to P_1_, P_2_, and P_3_, respectively, as in the main text. The positions of these peaks remain unaffected by the thickness variation of SnP_2_S_6_. (b) PL spectra from SnP_2_S_6_ samples with different thicknesses. The spectrum shapes were modulated by the thickness-dependent interference patterns and visibility within the 600–900 nm wavelength range.


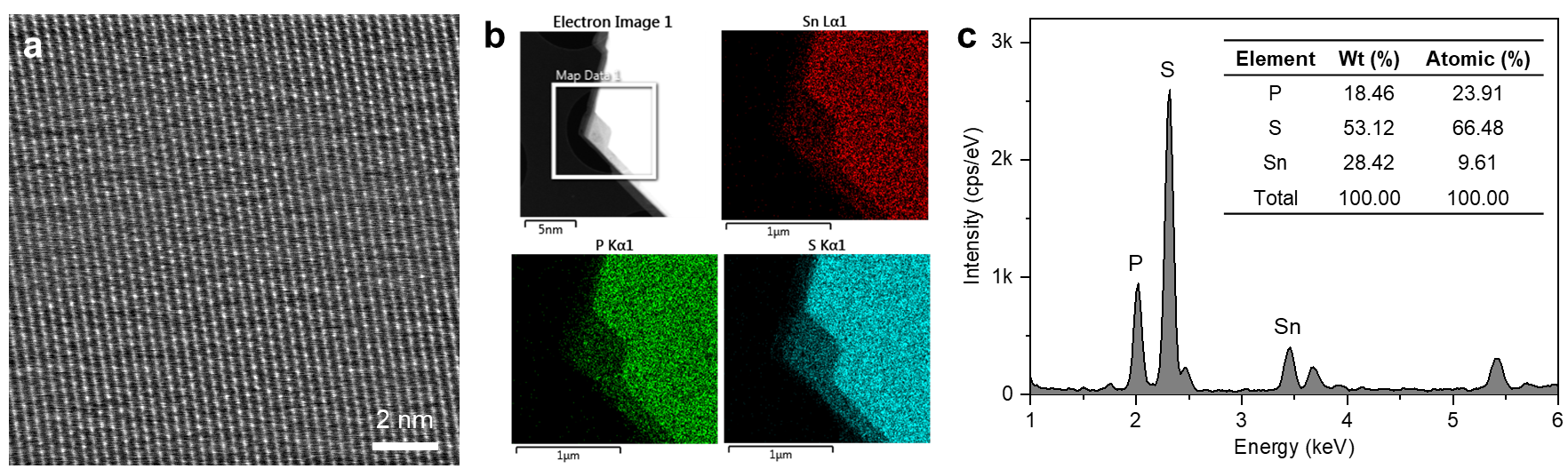


**FIGURE S3** Microscopic structural analysis of SnP_2_S_6_ thin film. (a) Low-magnification dark-field transmission electron microscopy image of a mechanically exfoliated SnP_2_S_6_ thin film. (b) Elemental mappings of our sample by energy-dispersive X-ray spectroscopy (EDS) for Sn (red), P (green), and S (blue) atoms, respectively, measured at the region indicated by the white edge square in the electron image. (c) EDS spectra of a SnP_2_S_6_ thin film, which reveals the atomic ratios of Sn, P, and S.


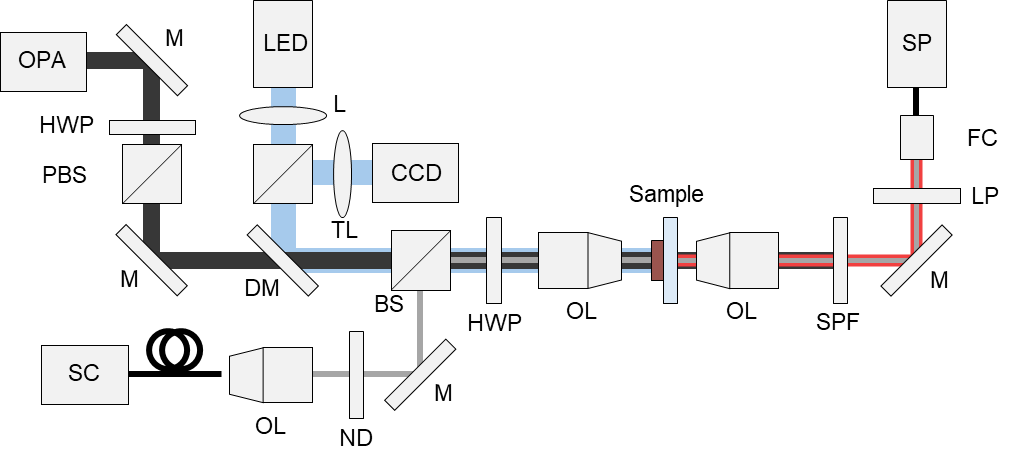


**FIGURE S4** Schematic diagram of experimental setup for broadband second-harmonic generation (SHG) spectroscopy and linear optical transmittance. OPA: optical parametric amplifier, M: mirror, HWP: half-wave plate, PBS: polarization beam splitter, SC: supercontinuum source, OL: objective lens, ND: neutral density filter, DM: dichroic mirror, TL: tube lens, L: lens, BS: beam splitter, SPF: short-pass filter, LP: linear polarizer, FC: fiber collimator, SP: spectrometer.


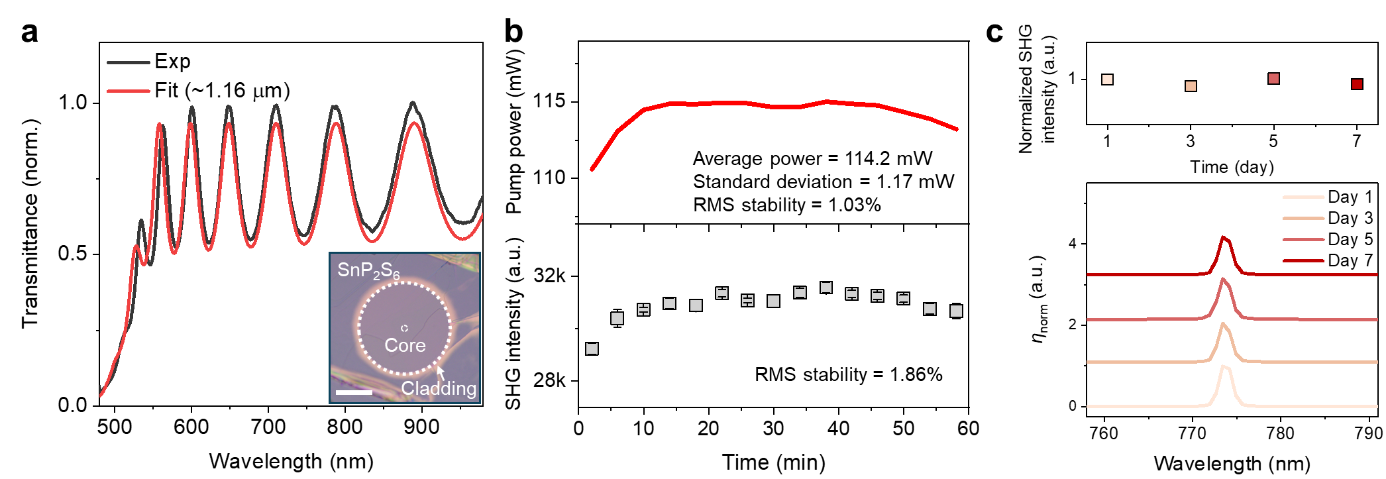


**FIGURE S5** Long-term stability of second-harmonic generation (SHG) under a continuous-wave (CW) pumping condition in an approximately 1.16 μm-thick SnP_2_S_6_-integrated fiber device. (a) Transmittance spectrum of the SnP_2_S_6_ film used for the fiber device, fitted using the Swanepoel method to determine the optical thickness. A minor spatial offset of the transferred film relative to the fiber core occurs during the integration process, leading to the slight discrepancy with the geometric thickness profile. Inset: Optical microscope image showing the surface of the fabricated SnP_2_S_6_-integrated fiber device, where the white scale bar at the left bottom corner denotes 50 μm. (b) Time-dependent SHG efficiency over a duration of 1 hour. The root-mean-square (RMS) fluctuations of the pump power and the SHG intensity were 1.03% and 1.86%, respectively, and show reasonably constant conversion efficiency. Here, RMS stability is defined as the standard deviation divided by the average value over the entire measurement period. (c) Long-term stability evaluated over 7 days: (upper) normalized SHG intensity tracked from Day 1 to Day 7, and (lower) the normalized conversion efficiency ($\eta_{norm}$) spectra.


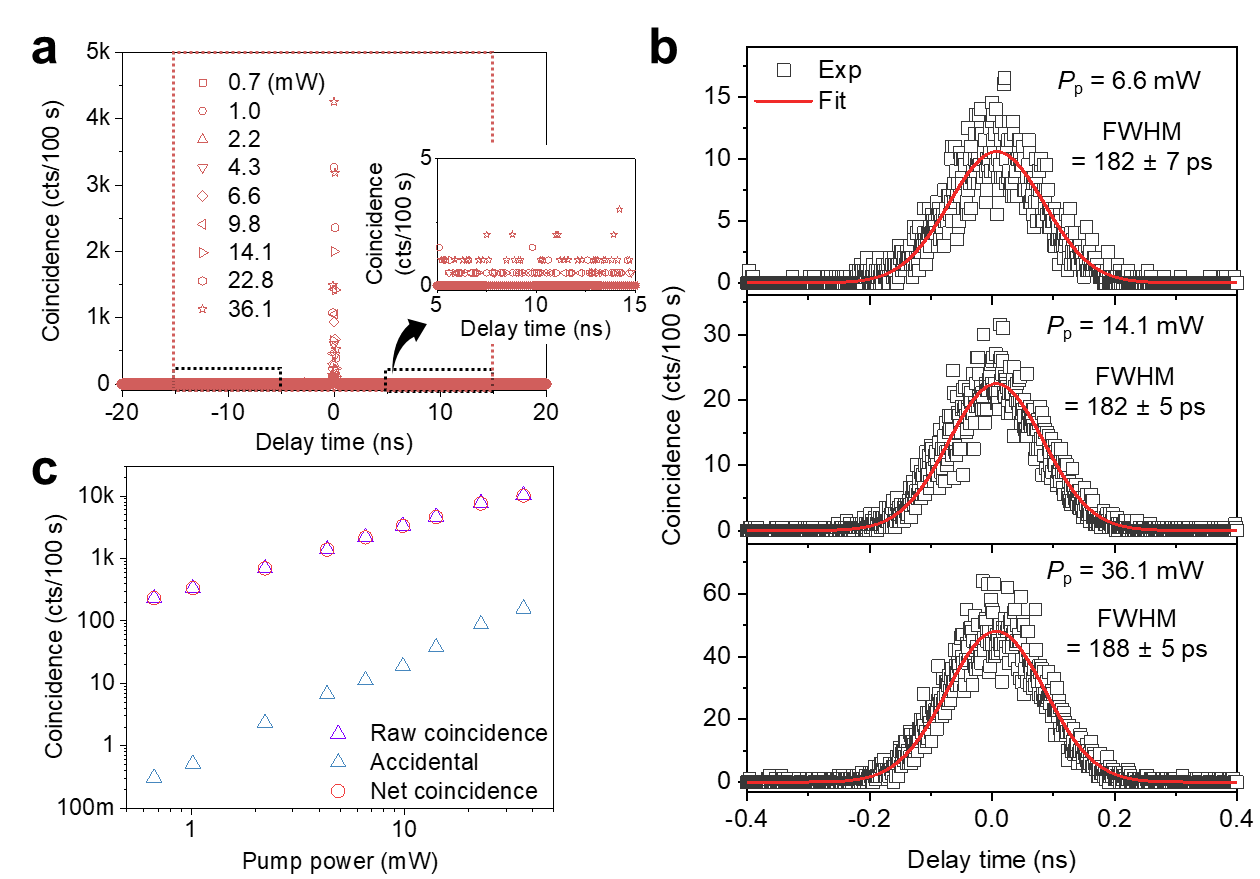


**FIGURE S6** Pump-power-dependent coincidence counts of the SnP_2_S_6_-integrated optical fiber device under a 100 s integration time using a 1500 nm long-pass filter. (a) Raw coincidence histogram, acquired with 1-ps time-bins, as a function of pump power (re-binned by 100 ps intervals for data presentation). All calculations required for the analysis are conducted within the region indicated by red dots (from -15 ns to 15 ns). The accidental coincidences are evaluated within the two regions (totaling 20 ns) marked by black dots, where one of them is shown as an inset. (b) Narrow temporal range of raw coincidence data fitted by a Gaussian function for pump powers of 6.6, 14.1, and 36.1 mW, exhibiting a reasonably constant FWHM ranging from 182 to 188 ps. (c) Raw, accidental, and net coincidence counts as a function of pump power. The coincidence window size for CAR measurement is set as 600 ps based on the results of (b).


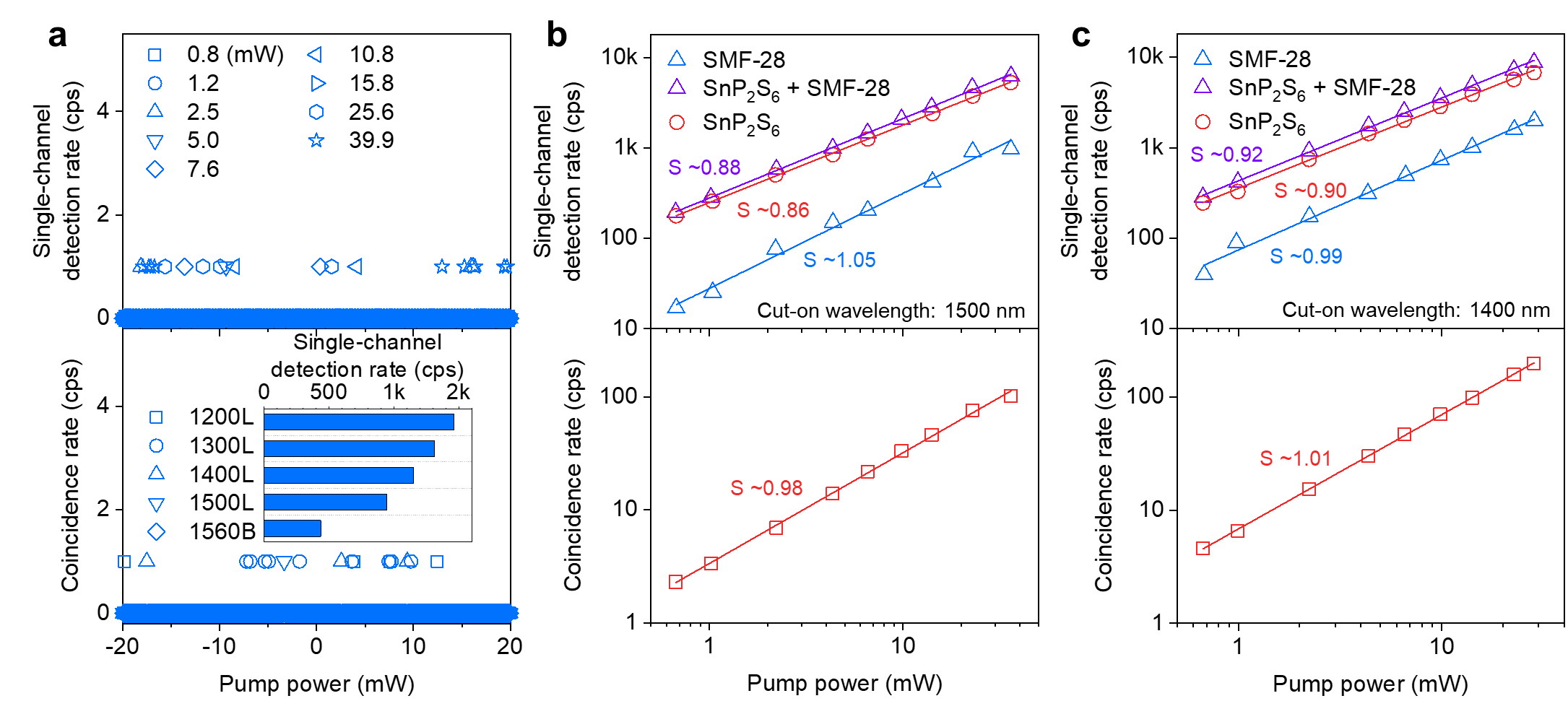


**FIGURE S7** Single and coincidence measurements for identifying background noise contributions. (a) Pump-power dependent coincidence counts measured with a 1500 nm cut-on wavelength long-pass filter (upper), and corresponding results using various spectral filters at 15.8 mW of average pump power of 782 nm continuous-wave laser (lower) in bare SMF-28 optical fiber. Inset: Single-channel detection rates for various filters. Both coincidence counts were obtained during the integration time of 60 s. (b,c) Pump-power-law scaling of single-channel (upper) and coincidence counts (lower) under 1500 nm and 1400 nm LPFs, respectively.


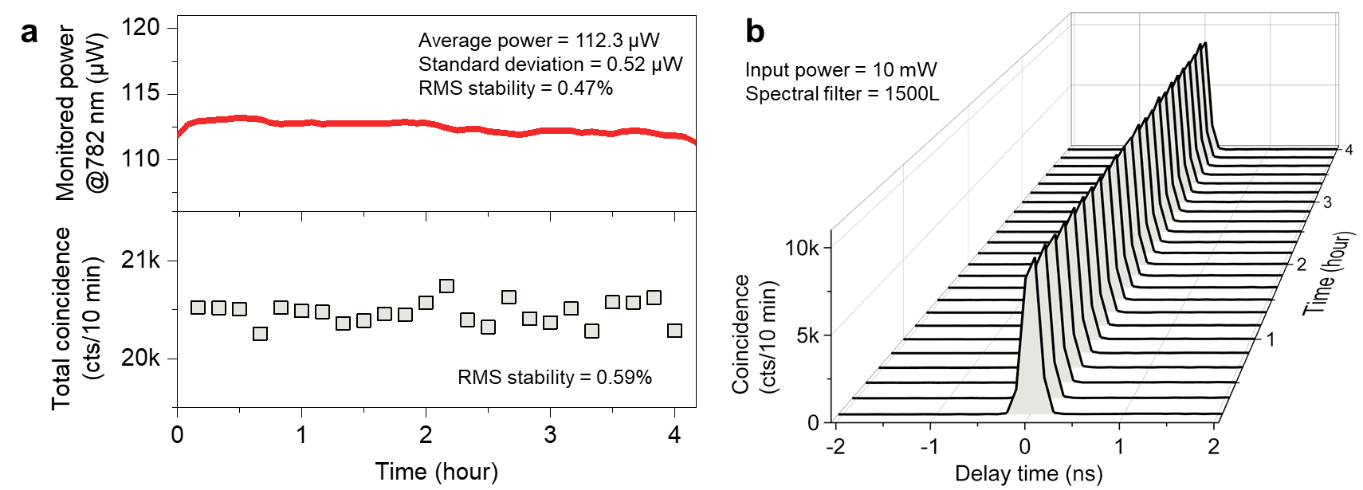


**FIGURE S8** Continuous-operation stability of SPDC in a 2.6 μm-thick SnP_2_S_6_-integrated fiber device. (a) Temporal stability comparison between the pump power (upper) and the total coincidence counts (integrated per 10 min; lower) over a total duration of 4 hours, demonstrating our system robustness under continuous pumping conditions. The RMS fluctuations of the pump power and the coincidence counts were 0.47% and 0.59%, respectively. (b) The raw coincidence histograms captured over time, which serve as the original data for the integrated counts presented in the lower graph of (a).

**
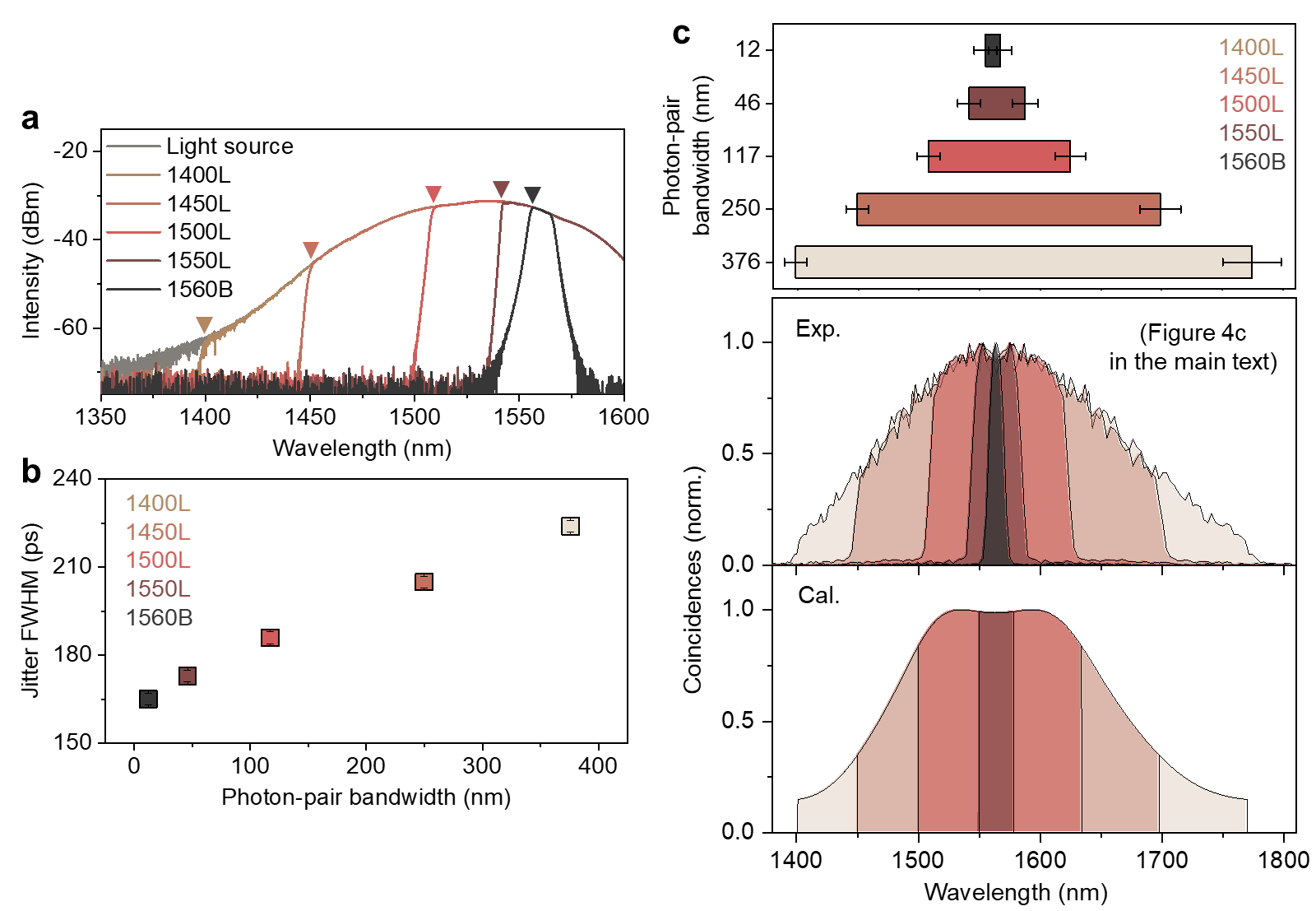
**

**FIGURE S9** Broadband SPDC behavior of the SnP_2_S_6_­-integrated fiber device. (a) Spectral profiles of superluminescent diodes (Thorlabs, S5FC1550P-A2) centered at 1550 nm with 90 nm bandwidth, filtered by various spectral filters. Downward triangles indicate the cut-on wavelengths 1398, 1449, 1508, 1541, and 1555 nm of the long-pass filters (Thorlabs 1400L, 1450L, 1500L, 1550L, 1560B), respectively. (b) FWHM of the coincidence peaks near zero-delay time plotted against the expected photon-pair bandwidth, derived from the spectral profiles in (a). (c) (top) Expected photon-pair bandwidth with error margins for various filtration conditions, extracted from the measured edge wavelength in (a), considering dispersion of SMF-28 fiber and the coincidence peak widths in (b). (middle) Experimentally measured SPDC spectra from the SnP_2_S_6_ film using fiber spectroscopy (same as Figure 4c in the main text). (bottom) Calculated SPDC spectra obtained by the numerical model described in the Experimental Section.


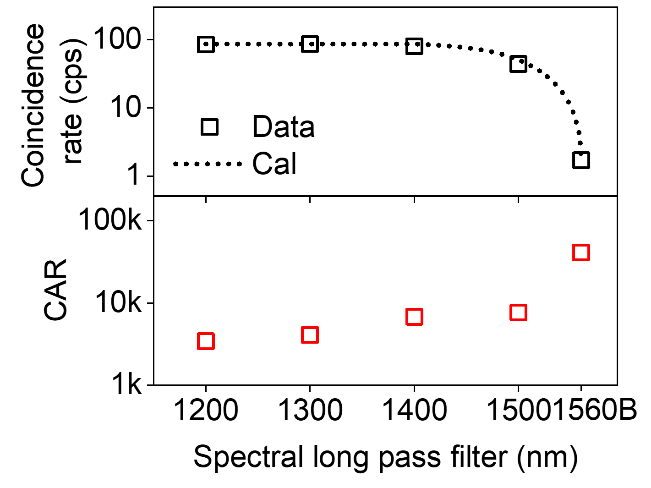


**FIGURE S10** Spectral filter-dependent coincidence rate and coincidence-to-accidental ratio (CAR) from the SnP_2_S_6_-integrated optical fiber. The experimental data (black squares in the upper graph) were well-fitted by the calculated coincidence rate $R_{coin}$ (black dotted line; Note S3). The number in the x-axis represents the cut-on wavelength (nm) of the long-pass filter, except for 1560, which is the center wavelength (nm) of the band-pass filter (BPF) with a bandwidth of 12 nm.


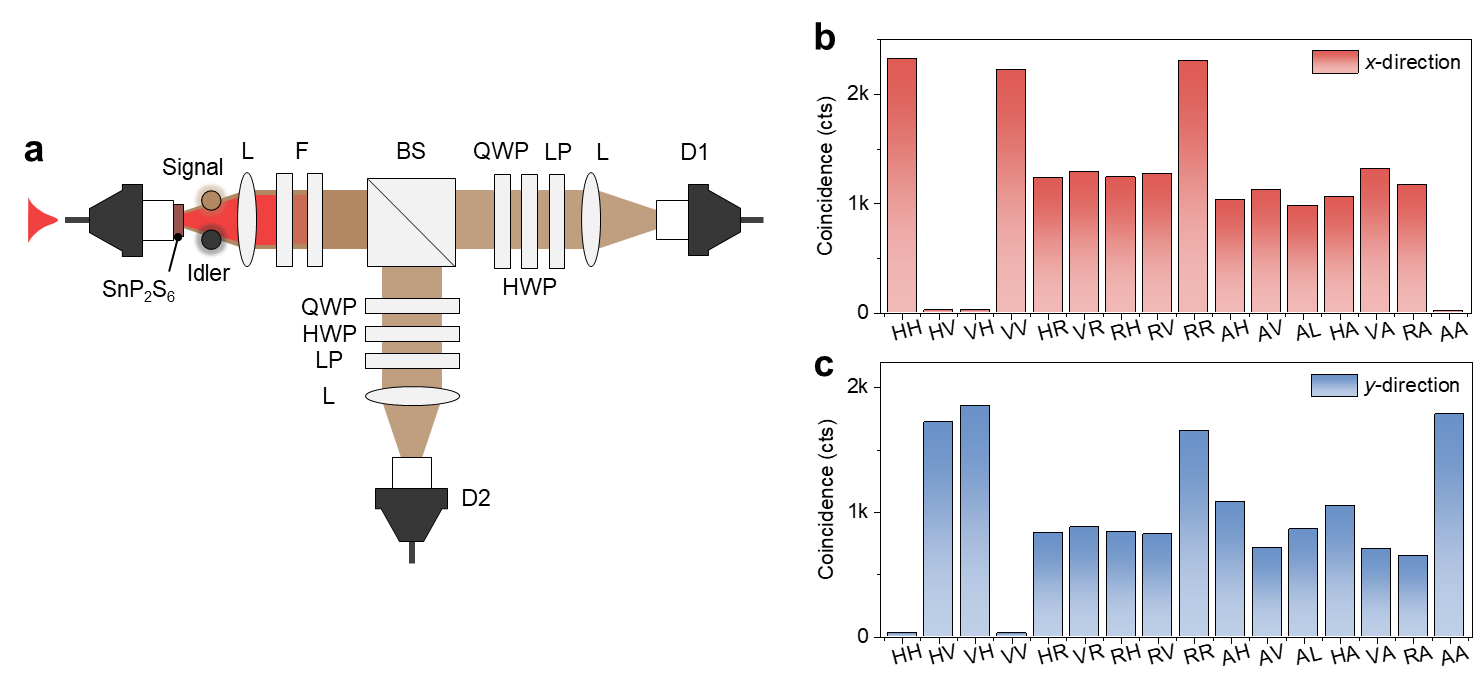


**FIGURE S11** Polarization correlations of entangled photon pairs produced by SnP_2_S_6_-integrated optical fiber. (a) Schematic of the experimental setup. Generated photon pairs are split into two paths by a nonpolarizing beam-splitter (BS); each path comprises a quarter-wave plate (QWP), a half-wave plate (HWP), a linear polarizer (LP), an aspheric lens (L), and a fiber-coupled single-photon detector (D1, D2). (b, c) Coincidence counts measured over 16 bases for pump polarization along the *x*-direction (b) and *y*-direction (c). F: 1500-nm long-pass filter.


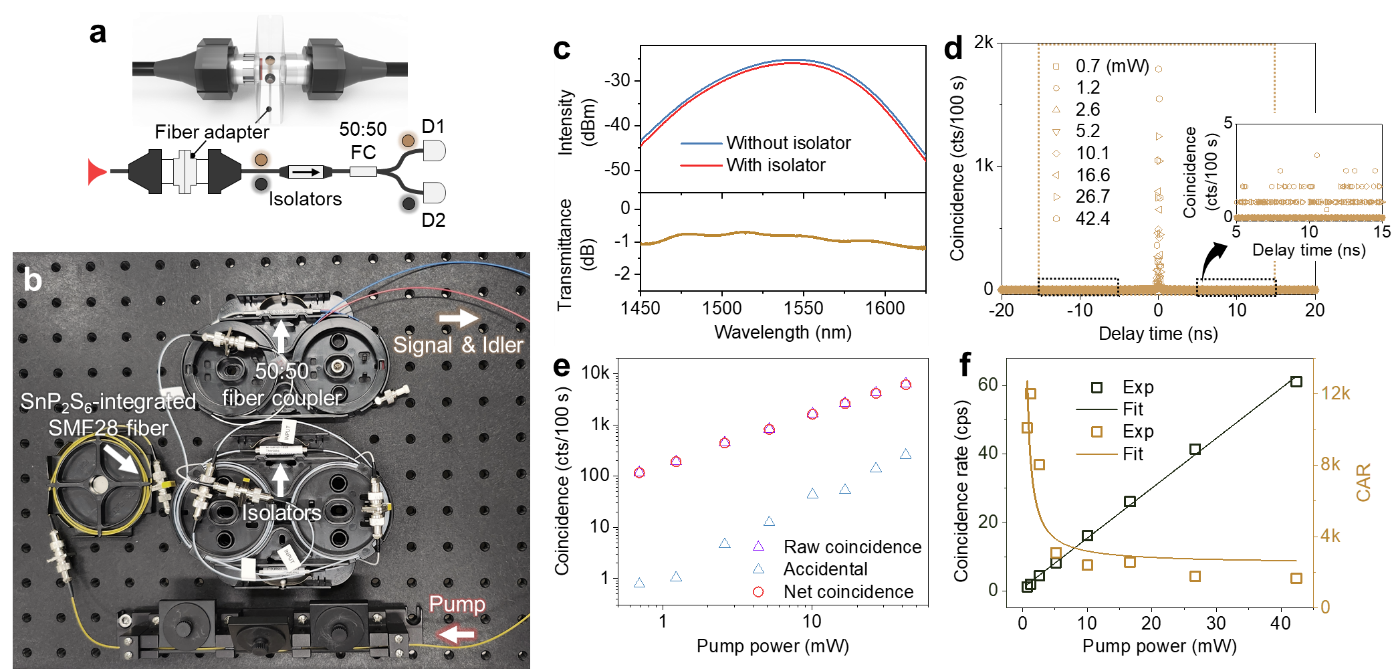


**FIGURE S12** Photon-pair generation characteristics in the proof-of-concept all-fiber configuration. (a, b) Schematic diagram and photograph of all-fiber SPDC experimental setup with our fiber device integrating SnP_2_S_6_ thin film, respectively. (c) Broadband transmission characteristics of the isolator used in (a, b). (d) Raw coincidence histogram, acquired with 1-ps time-bins, as a function of pump power (re-binned at 100-ps intervals for data presentation). Data treatments required for the analysis are conducted within the region indicated by yellow dots (from -15 ns to 15 ns). The accidental coincidences are evaluated within the two regions (totaling 20 ns) marked by black dots, where one of them is shown as an inset. (e) Raw, accidental, and net coincidence counts as a function of pump power. (f) Power-dependent average coincidence rate (black) and CAR (yellow).

**TABLE S1** Comparison of vdW material-based SPDC sources. QPM: quasi-phase matching, PPTMD: periodically-poled transition metal dichalcogenides, F: fidelity, C: concurrence, P: purity.


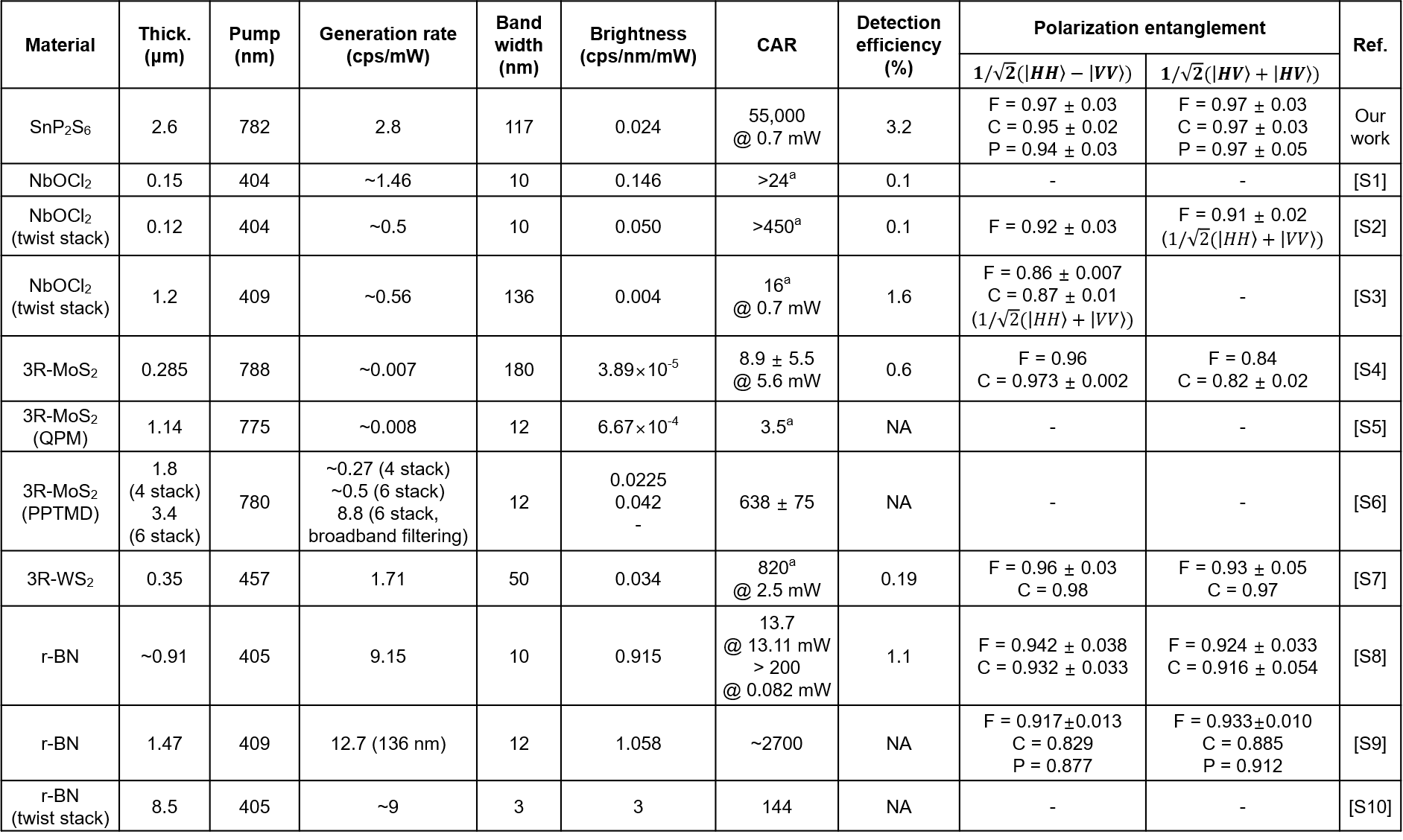


^a^Converted from the reported $g^{\left( 2 \right)}\left( 0 \right)$ using $\mathrm{CAR}\approx g^{\left( 2 \right)}\left( 0 \right)-1$ [S1, S11].

**Supporting Notes**

**NOTE S1** Extraction of Net Coincidence and Background Noise Analysis

The raw coincidence counts were obtained by integrating the histogram data over a 30 ns region centered at zero-delay time. To extract the net coincidence counts, the accidental coincidence counts were determined by evaluating the average background coincidence count per time-bin from two 10 ns off-peak flat regions and multiplying the average value by the number of time-bins that are within the temporal range of interest for coincidence counting. The final net coincidence counts and CAR were determined by subtracting these calculated accidental counts from the raw coincidence counts (Figure S6c and S12e).

Especially, to quantify the background noise contributions, we conducted photon-counting measurements as a function of pump power using a 1400 nm LPF to compare with the 1500 nm LPF results in the main text. This control experiment showed that the bare fiber single-channel counts roughly doubled with the 1400 nm LPF, consistently exhibiting near-unity slopes for both filters (blue triangles and fitted lines in upper graphs in Figures S7b and S7c). This suggests that the baseline noise is dominated by linear background single counts, which could arise from a combination of residual pump leakage and pump-induced emission in various fiber components (e.g., polarization controllers, couplers, and attenuators) prior to the sample. In contrast, for the SnP_2_S_6_-integrated fiber, the baseline-subtracted single-channel power-law scaling yielded sub-linear slopes below unity (0.86 for 1500 nm LPF and 0.90 for 1400 nm LPF, respectively; red circles and fitted lines in upper graphs in Figure S7b,c). These sub-linear slopes indicate the presence of material fluorescence undergoing power-dependent saturation, which can elevate accidental coincidences at shorter wavelengths. The coincidence counts for both filters scaled linearly with a near-unity slope (lower graphs in Figure S7b,c), verifying that the coincidence detection remains predominantly driven by the SPDC process, rather than being influenced by the single-channel background noise.

**NOTE S2** Dispersion Calibration and Spectral Resolution in Fiber Spectroscopy

To establish a time-delay-to-wavelength mapping, the system dispersion was calibrated based on the measurements of filter-edge wavelengths (Figure S9a). By fitting the edge tail of the 1400L, the zero-dispersion slope ($S_{0}$) and zero-dispersion wavelength ($\lambda_{0}$) of the SMF-28 fiber spool were determined to be 0.088 ps/nm^2^/km and 1310 nm, respectively. The wavelength-dependent dispersion $D(\lambda)$ was calculated via the dispersion formula for a non-dispersion-shifted fiber expressed as $D\left( \lambda\right)=\frac{\lambda S_{0}}{4}\left[ 1-\left( \frac{\lambda_{0}}{\lambda} \right)^{4} \right]$^S12^. The uncertainty due to timing jitter of the fiber spectroscopy measurement was experimentally determined from the FWHM of the coincidence peaks via Gaussian fitting (Figure S9b). This timing jitter leads to effective spectral resolution, which is shown as uncertainty margins of the measured bandwidths (top graph in Figure S9c). The agreement between these filter-extracted photon-pair bandwidths and the experimental/calculated spectral ranges consistently verifies the broadband nature of our characterized SPDC source (middle and bottom graphs in Figure S9c).

**NOTE S3** Numerical Modeling of SPDC Joint Spectral Intensity

The joint spectral intensity (JSI) is expressed as the product of the pump spectral envelope (Gaussian), which enforces energy conservation at the sum frequency of the signal and idler, and the phase-matching function:

$JSI\left( \lambda_{s},\lambda_{i} \right)\propto\exp\left[ -\frac{\left( \omega_{s}+\omega_{i}-\omega_{p} \right)^{2}}{2\sigma_{\omega}^{2}} \right]\times\mathrm{sinc}^{2} \left[ \frac{\Delta k_{z}\left( \lambda_{s},\lambda_{i} \right)\cdot L}{2} \right]\times T\left( \lambda_{s} \right)T\left( \lambda_{i} \right)$*,*

where $\sigma_{\omega}=\Delta\omega_{FWHM}/2\sqrt{2\ln2}$. Because the pump is a narrow-linewidth CW laser (linewidth ~3 kHz, corresponding to $\Delta\lambda\approx6 \mathrm{am}$), the Gaussian envelope reduces to a delta function $\delta\left( \omega_{s}+\omega_{i}-\omega_{p} \right)$. The wavelength dependence of the JSI is therefore governed by $\mathrm{sinc}^{2} \left[ \Delta k_{z}\left( \lambda_{s},\lambda_{i} \right)L/2 \right]$. Assuming a collinear configuration at normal incidence, the wavevector mismatch $\Delta k_{z}$ is defined as:

$\Delta k_{z}=\frac{2\pi n\left( \lambda_{p} \right)}{\lambda_{p}}-\frac{2\pi n\left( \lambda_{s} \right)}{\lambda_{s}}-\frac{2\pi n\left( \lambda_{i} \right)}{\lambda_{i}}$*.*

For the numerical modeling, second-order nonlinear susceptibility $\chi^{(2)}$ was fixed at 28 pm/V. This value corresponds to our experimentally measured $\chi^{(2)}$ at the fundamental wavelength of 1560 nm, which is close enough to the degenerate central wavelength (1564 nm) of photons under the 782-nm-pump condition. The complex refractive index profiles, $n(\lambda)$ and $k(\lambda)$, were determined using the ordinary-axis Sellmeier equation from Ref. S13, which is known to be valid over a broad spectral range (0.54–8.5 μm) and encompasses all relevant wavelengths without extrapolation. Since SnP_2_S_6_ is a semiconductor and the relevant photon energies lie well below its optical bandgap (~2.3 eV), single-photon absorption is negligible in the spectral range considered, thus the extinction coefficient $k(\lambda)$ was set to zero.

The transmittance $T(\lambda)$ for the end-face of the fiber/SnP_2_S_6_/air multilayer architecture was explicitly calculated using the standard Transfer Matrix Method (TMM) to incorporate Fabry-Pérot resonance effects. By accounting for these multilayer boundary conditions alongside the wavelength-dependent quantum efficiency of the single-photon detector, the film thickness *L* was self-consistently estimated to be 2.6 μm (slightly correcting the initial estimate of 2.7 μm derived solely from the peak SHG intensity).

To compute the coincidence rate for a given spectral window, the transmission window of a long-pass filter with a cut-on wavelength $\lambda_{cut}$ was modeled as a step function:

$C\left( \lambda\right)=\left\{ \begin{aligned} 1 \lambda\geq\lambda_{cut} \\ 0 \lambda<\lambda_{cut} \end{aligned} \right.$.

The filtered JSI is then defined as $R\left( \lambda_{s}, \lambda_{i} \right)=JSI\left( \lambda_{s},\lambda_{i} \right)\cdot C\left( \lambda_{s} \right)\cdot C\left( \lambda_{i} \right)$, and the total coincidence rate ($R_{coin}$) was obtained by numerically integrating the filtered JSI weighted by the detector efficiency over the signal and idler wavelengths:

$R_{coin}\propto\iint RQ\left( \lambda_{s} \right)Q\left( \lambda_{i} \right)d\lambda_{s}d\lambda_{i}$,

where $Q\left( \lambda\right)$ represents the wavelength-dependent quantum efficiency of the single-photon detector.

**References**

S1. Guo, Q., Qi, X.-Z., Zhang, L., *et al.*, “Ultrathin quantum light source with van der Waals NbOCl2 crystal,” *Nature* **2023** *613*, 53–59, <https://doi.org/10.1038/s41586-022-05393-7>.

S2. Guo, Q., Wu, Y.-K., Zhang, D., *et al.*, “Polarization entanglement enabled by orthogonally stacked van der Waals NbOCl2 crystals,” *Nature Communications* **2024** *15*, 10461, <https://doi.org/10.1038/s41467-024-54876-w>.

S3. Kallioniemi, L., Lyu, X., He, R., *et al.*, “Van der Waals engineering for quantum-entangled photon generation,” *Nature Photonics* **2025** *19*, 142–148, <https://doi.org/10.1038/s41566-024-01545-5>.

S4. Weissflog, M. A., Fedotova, A., Tang, Y., *et al.*, “A tunable transition metal dichalcogenide entangled photon-pair source,” *Nature Communications* **2024** *15*, 7600, <https://doi.org/10.1038/s41467-024-51843-3>.

S5. Tang, Y., Sripathy, K., Qin, H., *et al.*, “Quasi-phase-matching enabled by van der Waals stacking,” *Nature Communications* **2024** *15*, 9979, <https://doi.org/10.1038/s41467-024-53472-2>.

S6. Trovatello, C., Ferrante, C., Yang, B., *et al.*, “Quasi-phase-matched up- and down-conversion in periodically poled layered semiconductors,” *Nature Photonics* **2025** *19*, 291–299, <https://doi.org/10.1038/s41566-024-01602-z>.

S7. Feng, J., Wu, Y.-K., Duan, R., *et al.*, “Polarization-entangled photon-pair source with van der Waals 3R-WS2 crystal,” *eLight* **2024** *4*, 16, <https://doi.org/10.1186/s43593-024-00074-6>.

S8. Liang, H., Gu, T., Lou, Y., *et al.*, “Tunable polarization entangled photon-pair source in rhombohedral boron nitride,” *Science Advances* **2025** *11*, eadt3710, <https://doi.org/10.1126/sciadv.adt3710>.

S9. Lyu, X., Kallioniemi, L., Hong, H., *et al.*, “A tunable entangled photon-pair source based on a Van der Waals insulator,” *Nature Communications* **2025** *16*, 1899, <https://doi.org/10.1038/s41467-025-56436-2>.

S10. Lin, K., Yao, G., Shao, J., *et al.*, “Nonlinear phase-matched van der Waals crystals integrated on optical fibres,” *Nature Materials* **2026** *25*, 581–587, <https://doi.org/10.1038/s41563-025-02461-x>.

S11. Okoth, C., Cavanna, A., Santiago-Cruz, T., Chekhova, M. V., “Microscale Generation of Entangled Photons without Momentum Conservation,” *Physical Review Letters* **2019** *123*, 263602, <https://doi.org/10.1103/PhysRevLett.123.263602>.

S12. Keiser, G. “Optical fiber communications.” *New York: McGraw-Hill* **2010** (4^th^ ed.), pp. 102-150.

S13. He, J., Lee, S. H., Naccarato, F., *et al.*, “SnP2S6: A Promising Infrared Nonlinear Optical Crystal with Strong Nonresonant Second Harmonic Generation and Phase-Matchability,” *ACS Photonics* **2022** *9*, 1724–1732, <https://doi.org/10.1021/acsphotonics.2c00131>.
